# The influence of virtual reality technology on upper limb motor function in subacute stroke: a systematic review and meta-analysis

**DOI:** 10.7717/peerj.21073

**Published:** 2026-04-16

**Authors:** Changjin Wu, Jinghan Zhu, Hongyu Pan, Yi Zhou, Jiahuan Wang, Xin Zhou, Lidan Zhang, Fanping Kong

**Affiliations:** Sanya Rehabilitation and Recuperation Center, Sanya, China

**Keywords:** Immersive virtual reality, Non-immersive virtual reality, Subacute stage hemiparesis, Motor function, Upper extremity, Fugl-meyer assessment, Randomized controlled trial, Meta-analysis

## Abstract

**Objective:**

This study aimed to evaluate the efficacy of virtual reality (VR) in upper limb rehabilitation in subacute stroke, including the Fugl-Meyer Assessment Upper Extremity Scale (FMA-UE), Box and Block Test (BBT), and Action Research Arm Test (ARAT). Subgroup analysis was performed to explore the optimal intervention parameters for VR treatment.

**Methods:**

PubMed, Embase, Cochrane Library, and Web of Science were thoroughly searched for randomized controlled trials (RCTs) on the use of VR for the rehabilitation of upper limb dysfunction in post-stroke hemiplegic patients until December 2025. Eligible studies were analyzed via RevMan 5.3 statistical software, with study quality assessed by the AOS scoring system, and data analysis performed using Stata 15.

**Results:**

Fifteen studies were encompassed, comprising 612 cases overall, with 318 cases in the experimental group and 294 cases in the control group. VR rehabilitation was superior to traditional rehabilitation in FMA-UE score (SMD = 0.68, 95% CI [0.30–1.05], *P* = 0.000), but there was no significant difference in BBT (SMD = 0.44, 95% CI [−0.02–0.90], *P* = 0.058), ARAT (SMD = 0.63, 95% CI [−0.01–1.27], *P* = 0.052) scores (*P* > 0.05). Subgroup analysis showed that FMA-UE scores were significantly improved when VR training exceeded 30 minutes/session, the frequency was 4–5 days/week, and the total intervention time was 3–4 weeks. Immersive VR training showed advantages in FMA-UE, BBT, and ARAT scores. According to the Cochrane 1.0 quality assessment system, the overall methodological quality of this study was rated as moderate. A total of eight follow-up studies were included: Four of the follow-up studies showed differences in outcome measures between the two groups.

**Conclusion:**

VR may have advantages in promoting FMA-UE scores in the upper limbs of patients with subacute stroke. VR training with more than 30 minutes per session, more than 4 days per week, and a total intervention time of more than 3 weeks may be more conducive to the recovery of upper limb function (ULF) in subacute stroke. However, the efficacy of VR in improving fine motor skills and dexterity of the hand, assessed by the BBT and ARAT, remains to be elucidated. Although these findings are informative, the conclusions should be interpreted with caution in consideration of the study’s limitations. Registry number: CRD42025610757.

## Introduction

Stroke, the second leading cause of death globally, features high incidence, disability, recurrence, and economic impact (Zhao et al., 2023). About 70% of stroke survivors experience upper limb paralysis, hindering daily activities, especially those requiring coordination or fine motor skills, rendering upper limb function (ULF) recovery a priority in rehabilitation (Meadmore et al., 2019).

Conventional interventions such as motor imagery therapy, mirror therapy, constraint-induced movement therapy, and active sensory therapy have demonstrated substantial clinical efficacy in upper limb rehabilitation and are all effective in promoting the recovery of motor function in the paretic upper extremity. However, traditional rehabilitation is often lengthy, repetitive, and monotonous, resulting in low patient engagement (Sun et al., 2024; Wang et al., 2014). Virtual reality (VR) has gained popularity in neurorehabilitation, providing interactive environments with multisensory feedback and integration with devices like treadmills and robotic arms (Choi & Paik, 2018; Hao et al., 2022). They can integrate with devices like treadmills, bionic gloves, or robotic arms. Early VR was non-immersive and used 2D environments for interaction *via* mouse or joystick. Semi-immersive VR enables visual exploration, while immersive VR offers a lifelike experience through multisensory interaction. VR also supports customizable rehabilitative training games with adjustable intensity and personalized protocols (García-Betances et al., 2015; Kim, Darakjian & Finley, 2017; Wu, Ma & Ren, 2020). VR training enhances engagement and high-intensity exercise adherence more than traditional rehabilitation, significantly ameliorating outcomes (Chatterjee et al., 2022).

Although VR’s role in chronic stroke rehabilitation is well-researched (Song & Lee, 2021; Wu et al., 2021), the subacute phase of stroke recovery is particularly critical, and evidence regarding the efficacy of VR-based interventions for upper limb rehabilitation during this stage remains inconsistent (Donati et al., 2025). In recent years, advances in VR technology have facilitated the development of game-based hand function rehabilitation programs, but their effects on improving fine motor performance and dexterity of the affected hand, as assessed by the Action Research Arm Test (ARAT) and Box and Block Test (BBT), warrant further investigation. Moreover, the reliability of existing conclusions is limited by generally small sample sizes and substantial heterogeneity in intervention protocols. In addition, few studies have simultaneously employed the three core upper limb outcome measures, Fugl-Meyer Assessment Upper Extremity Scale (FMA-UE), BBT, and ARAT, thereby constraining a comprehensive evaluation of true intervention effects. Accordingly, this study aims to conduct a meta-analysis to systematically evaluate the effects of VR interventions, compared with conventional rehabilitation, on ULF recovery in patients with subacute stroke, and to explore evidence-based optimal VR intervention parameters through subgroup analyses, thereby guiding clinical practice.

## Materials and Methods

### Literature search

An online retrieval was performed by December 2025 for randomized controlled trials (RCTs) evaluating the utilization of VR therapy for the rehabilitation of ULF in subacute stroke patients across the PubMed, Embase, Cochrane, and Web of Science databases. The keywords used in the search were “VR” and “stroke”. The search strategy is detailed in the supplementary materials Table S1.

### Eligibility criteria

#### Inclusion criteria

The study adopted the PICOS framework: (1) Population: Stroke patients diagnosed per WHO standards, confirmed by CT or MRI, with onset between 7 days and 6 months (Bernhardt et al., 2017) before enrollment; (2) Intervention: VR-based rehabilitation; (3) Comparison: Conventional physical rehabilitation, such as range-of-motion exercises, stretching, strength training, and activities of daily living training, and occupational therapy, with other consistent treatments; (4) Outcomes: The primary outcome was FMA-UE, which evaluates upper limb coordination, range of motion, and motor control in stroke patients. Secondary outcomes encompassed BBT, which assesses manual dexterity and fine motor skills, and ARAT, which evaluates grasp, grip, pinch, and gross arm movements. (5) Study Design: RCTs.

#### Exclusion criteria

(1) Non-experimental studies, case reports, reviews, commentaries, systematic reviews, and qualitative research. (2) Duplicates, studies on acute or chronic stroke patients, or those with inconsistent outcome measures. (3) Incomplete data (*e.g.*, lacking means and standard deviations) affecting meta-analysis integrity.

### Data extraction

YZ and JHW checked the literature and extracted data independently. Titles, abstracts, and full-text articles were reviewed. For potentially eligible studies with disagreements, opinions from relevant experts were sought, and the full-text papers were obtained for further review. The quality of the eligible studies was independently assessed by two reviewers (*XZ*, and *CJW*) using the Joanna Briggs Institute (JBI) critical appraisal tools (Bossuyt et al., 2015). The eligibility criteria were adhered to during screening. Data on relevant outcomes were extracted. A cross-check of the extracted data was performed to ensure consistency across the reviewers. Extracted information encompassed (1) Basic study information: First author, country, as well as year of publication; (2) Study population, intervention, and duration; (3) Key elements for risk of bias evaluation; (4) Outcome: FMA-UE, BBT, and ARAT.

### Quality assessment

FPK and LDZ assessed the risk of bias *via* the Cochrane1.0 Collaboration (Cumpston et al., 2019), categorizing it as low, unclear, or high. JHZ resolved disagreements. They examined implementation, observational, selective (randomization and allocation), follow-up, reporting, and other biases. Low risk indicated high quality, unclear risk suggested moderate bias, and high risk implied poor quality.

### Statistical analysis

Data processing and statistical analysis were enabled by RevMan 5.3. Study heterogeneity was evaluated with observation of the data extraction table. In such cases, potential sources of heterogeneity were investigated by reviewing data extraction, clinical interventions, study designs, and subgroup analyses by intervention methods, duration, frequency, and total time. A fixed-effects model was used if *P* > 0.1 and I^2^ < 50%, indicating study homogeneity. Conversely, a random-effects model was applied if *P* ≤ 0.1 and I^2^ ≥ 50%, indicating significant heterogeneity. Sensitivity analyses were performed by excluding individual studies sequentially. Continuous variables were expressed as standardized mean differences (SMDs) with 95% confidence intervals (CIs), and *P* < 0.05 denoted statistical significance. Publication bias was assessed *via* funnel plots.

## Results

### Literature search process and results

**Figure 1:**
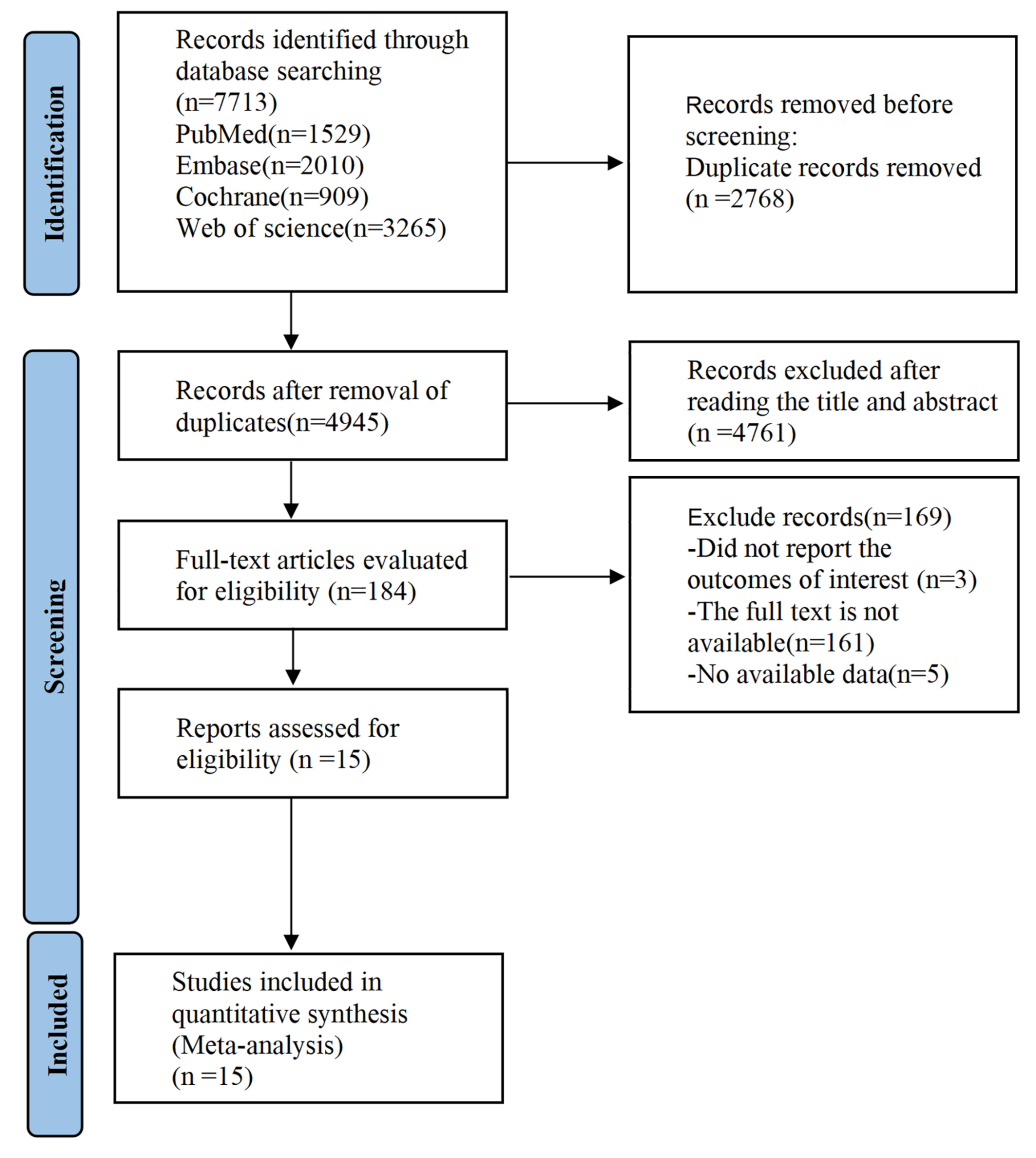
The literature screening process.

A total of 7,479 relevant articles were retrieved. After 2,622 duplicate records and 4,682 studies based on title and abstract were excluded, 175 articles remained. Through a full-text review, 15 eligible studies were included. These studies comprised 627 subjects in total, with 323 in the experimental cohort and 304 in the control cohort. Our literature selection steps and results are depicted in Fig. 1.

### Basic characteristics of the included studies

The study analyzed 15 RCTs with 627 hemiparetic subacute stroke patients. 13 studies focused on stroke durations of 7 days to 3 months, and two on 7 days to 6 months. Participants underwent both immersive and non-immersive VR therapy for two to six weeks, with sessions of 30–60 min, three to five times weekly. Immediately after completion of the full VR treatment cycle, outcome measures were assessed. The main outcome was FMA-UE (Table 1, Table 2) (Amin et al., 2024; Brunner et al., 2017; Chen et al., 2022; Cumpston et al., 2019; Huang et al., 2024; Ikbali Afsar et al., 2018; Jo et al., 2024; Kim et al., 2018; Lee & Chun, 2014; Leng et al., 2022; Mekbib et al., 2021; Rodríguez-Hernández et al., 2021; Rodríguez-Hernández et al., 2023; Shin et al., 2022; Yin et al., 2014).

**Table 1:** Characteristics of studies in our meta-analysis.

Study	Year	Region	Sample size (M/F)	Gender (M/F)	Mean age (years)	Intervention	Outcomes	Type of VR
Jo et al. (2024)	2024	Korea	imVR: 15 Control: 15	15/15	imVR: 51.73 ± 13.63 Control: 47.13 ± 13.91	30 min, 3 days a week, for 4 weeks	F1, F2	360° imVR+Mirror therapy
Huang et al. (2024)	2024	China	imVR: 15 Control: 15	24/16	imVR: 63.3 ± 14.3 Control: 65.1 ± 6.1	30 min, 5 days a week, for 3 weeks	F2	imVR
Amin et al. (2024)	2024	Pakistan	imVR: 26 Control: 26	34/18	imVR: 51.8 ± 12.9 Control: 49.8 ± 9.9	35 min, 4 days a week, for 6 weeks	F1, F2, F3	imVR+visual feedback
Rodríguez-Hernández et al. (2023)	2023	Spain	S-VR: 23 Control: 20	35/8	VR: 62.6 ± 13.5 Control: 63.6 ± 12.2	50 min, 5 days a week, for 3 weeks	F2, F3	specifc VR+glove
Chen et al. (2022)	2022	China	Non-VR: 18 Control: 18	20/16	VR: 57.8 ± 8.4 Control: 59.12 ± 11.62	45 min, 5 days a week, for 2 weeks	F2, F3	Non-VR+ecoskeleton arm
Leng et al. (2022)	2022	China	Non-VR: 31 Control: 26	43/14	VR: 59.25 ± 10.7 Control: 59.12 ± 11.62	30 min, 5 days a week, for 3 weeks	F2	Non-VR+Kinect sensor
Shin et al. (2022)	2022	Korea	Non-VR: 20 Control: 16	17/19	VR: 57.±12.78 Control: 63.69 ± 8.58	30 min, 5 days a week, for 4 weeks	F2	Non-VR+Smart glove
Mekbib et al. (2021)	2021	China	imVR: 12 Control: 11	17/6	VR: 52.17 ± 13.26 Control: 61.00 ± 7.69	60min, 4 days a week, for 2 weeks	F2	imVR+Mirror therapy
Rodríguez-Hernández et al. (2021)	2021	Spain	Non-VR: 23 Control: 20	35/8	VR: 62.6 ± 13.5 Control: 63.6 ± 12.2	50 min, 5 days a week, for 3 weeks	F2	Non-VR+Kinect sensor
Ikbali Afsar et al. (2018)	2018	Turkey	Non-VR: 19 Control: 16	20/15	VR: 69.92 ± 8.55 Control: 63.44 ± 15.73	30 min, 5 days a week, for 4 weeks	F1, F2	Non-VR+games
Kim et al. (2018)	2018	Korea	Non-VR: 12 Control: 11	17/6	VR: 56.78.±17.8 Control: 57.2 ± 15	30 min, 5 days a week, for 2 weeks	F1, F2	Non-VR+Kinect sensor
Brunner et al. (2017)	2017	Norway	Non-VR: 62 Control: 58	77/43	VR: 62 ± 16.5 Control: 62 ± 11.5	60min, 4 days a week, for 4 weeks	F1, F3	Non-VR+Sensor-equipped glove
Lee & Chun (2014)	2014	Korea	Non-VR: 20 Control: 19	19/20	VR: 60.6 ± 14.1 Control: 60.3 ± 11.3	30 min, 5 days a week, for 3 weeks	F1, F2	Non-VR +glove
Yin et al. (2014)	2014	Singapore	Non-VR: 11 Control: 12	16/7	VR: 62 ± 5.5 Control: 56 ± 3.75	30 min, 5 days a week, for 2 weeks	F2, F3	Non-VR+electromagnetic sensors
Saposnik et al. (2010)	2010	Canada	Non-VR: 11 Control: 11	14/8	VR: 55.3 ± 9.25 Control: 67.3 ± 7.75	60 min, 4 days a week, for 2 weeks	F1	Non-VR+gaming system

**Table 2:** Interventions in the experimental and control groups.

Study	Experimental group	Control group
Jo et al. (2024)	Immersive VR (mirror therapy) + conventional rehabilitation	Conventional rehabilitation (neurodevelopmental treatment, muscle strengthening exercises, and range-of-motion exercises)
Huang et al. (2024)	Immersive VR + conventional rehabilitation	Conventional rehabilitation + occupational therapy
Amin et al. (2024)	Immersive VR + conventional rehabilitation	Conventional rehabilitation (range-of-motion, stretching, resistance, and strengthening exercises) + occupational therapy
Rodríguez-Hernández et al. (2023)	SVR system + conventional rehabilitation	Conventional rehabilitation (exercise therapy, range-of-motion, and resistance exercises) + occupational therapy
Chen et al. (2022)	Non-immersive VR	Occupational therapy (task-oriented motor training)
Leng et al. (2022)	Non-immersive VR + conventional rehabilitation	Conventional rehabilitation (range-of-motion exercises, muscle strengthening, functional training, neurodevelopmental therapy, proprioceptive neuromuscular facilitation, and electrotherapy)
Shin et al. (2022)	VR (repetitive task-oriented training delivered via an intelligent glove digital system) + occupational therapy	Occupational therapy
Mekbib et al. (2021)	Immersive VR + occupational therapy	Occupational therapy (distal and proximal upper limb functional exercises, activities of daily living, balance control, and gait training)
Rodríguez-Hernández et al. (2021)	SVR (real-world exposure therapy) + conventional rehabilitation	Conventional rehabilitation (exercise therapy; range-of-motion exercises; slope and stair training; resistance exercises; and simulated biomechanical tasks for the shoulder, wrist, and fingers) + occupational therapy
Ikbali Afsar et al. (2018)	VR + conventional rehabilitation	Conventional rehabilitation (proprioceptive neuromuscular facilitation and neurodevelopmental techniques; static and dynamic control; balance skills; weight shifting; and activities of daily living)
Kim et al. (2018)	VR + occupational therapy	Sham VR + occupational therapy
Brunner et al. (2017)	VR	Conventional rehabilitation (various grasp patterns and selective gross motor and dexterity training of the hand)
Lee & Chun (2014)	VR + conventional rehabilitation	Conventional rehabilitation (physical therapy and cognitive therapy) + occupational therapy
Yin et al. (2014)	VR + conventional rehabilitation	Conventional rehabilitation (stretching, strengthening, balance, gait, and functional training) + occupational therapy
Saposnik et al. (2010)	VR + conventional rehabilitation	Recreational therapy + conventional rehabilitation (physical therapy) + occupational therapy

### Risk of bias assessment

The overall quality of eligible studies was relatively high. All of them were RCTs and mentioned randomization. Every trial was rated as having a low risk of bias. Outcome data were complete for all studies, with no proof of selective reporting. The degree of risk was still unknown because other possible risks of bias were not discussed. Figure S1 displays the proportion of bias risk items in eligible studies, and Fig. S2 presents the risk of bias summary.

### Meta-analysis results

#### Influence of VR technology on FMA-UE

**Figure 2:**
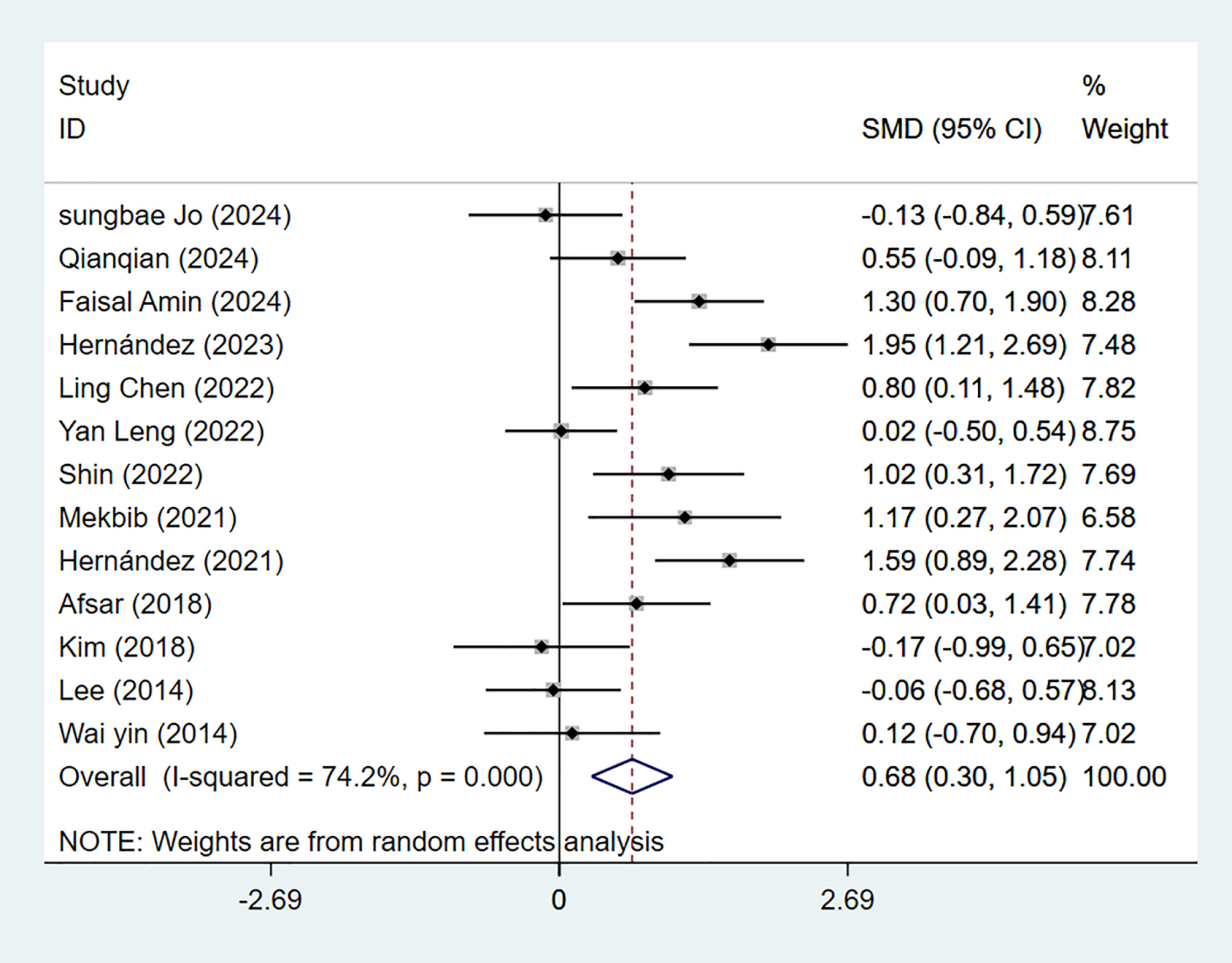
Forest plot of FMA-UE.

Thirteen studies employed FMA-UE to evaluate upper limb motor function in 459 patients with subacute stroke. Meta-analysis revealed a statistically significant effect (*P* = 0.000) with substantial heterogeneity among the included studies (*I*^2^ = 74.2%). Subgroup analysis by the intervention type (VR *vs.* CT) and subsequent sensitivity analysis by sequential exclusion of individual studies demonstrated that the VR intervention group exhibited more improvement in FMA-UE scores than the CT group (95% CI [0.30–1.05], *P* = 0.000), with a moderate effect size (SMD = 0.68). No significant source of bias was identified (Fig. 2 and Fig. S3).

#### Influence of VR on BBT

Seven studies utilized BBT to assess hand dexterity and fine motor skills in 315 patients with subacute stroke. The pooled analysis indicated statistical significance (*P* = 0.002) and considerable heterogeneity across studies (*I*^2^ = 71.6%). Subgroup analysis by intervention type (VR *vs.* CT), combined with stepwise exclusion of studies, revealed that VR training did not demonstrate a statistically significant advantage over conventional rehabilitation in improving BBT scores (95% CI [−0.02–0.90], *P* = 0.058), despite a moderate effect size (SMD = 0.44). No significant risk of bias was detected (Figs. S4–S5).

#### Influence of VR technology on ARAT

Five studies applied the Action Research Arm Test (ARAT) to evaluate ULFs, specifically grasp, grip, and gross arm movements, in 274 patients during the subacute phase of stroke. Meta-analysis indicated significant heterogeneity (*P* = 0.000, *I*^2^ = 82.6%). Subgroup analysis by intervention type (VR *vs.* CT) and sensitivity testing *via* stepwise study exclusion showed that VR training did not result in statistically significant improvement in ARAT scores compared to conventional rehabilitation (95% CI [−0.01–1.27], *P* = 0.052), although a moderate effect size was observed (SMD = 0.63). No major source of bias was found (Figs. S6–S7).

### Subgroup analysis results

#### Subgroup analysis of FMA-UE

##### Subgroup analysis by intervention method.

**Figure 3:**
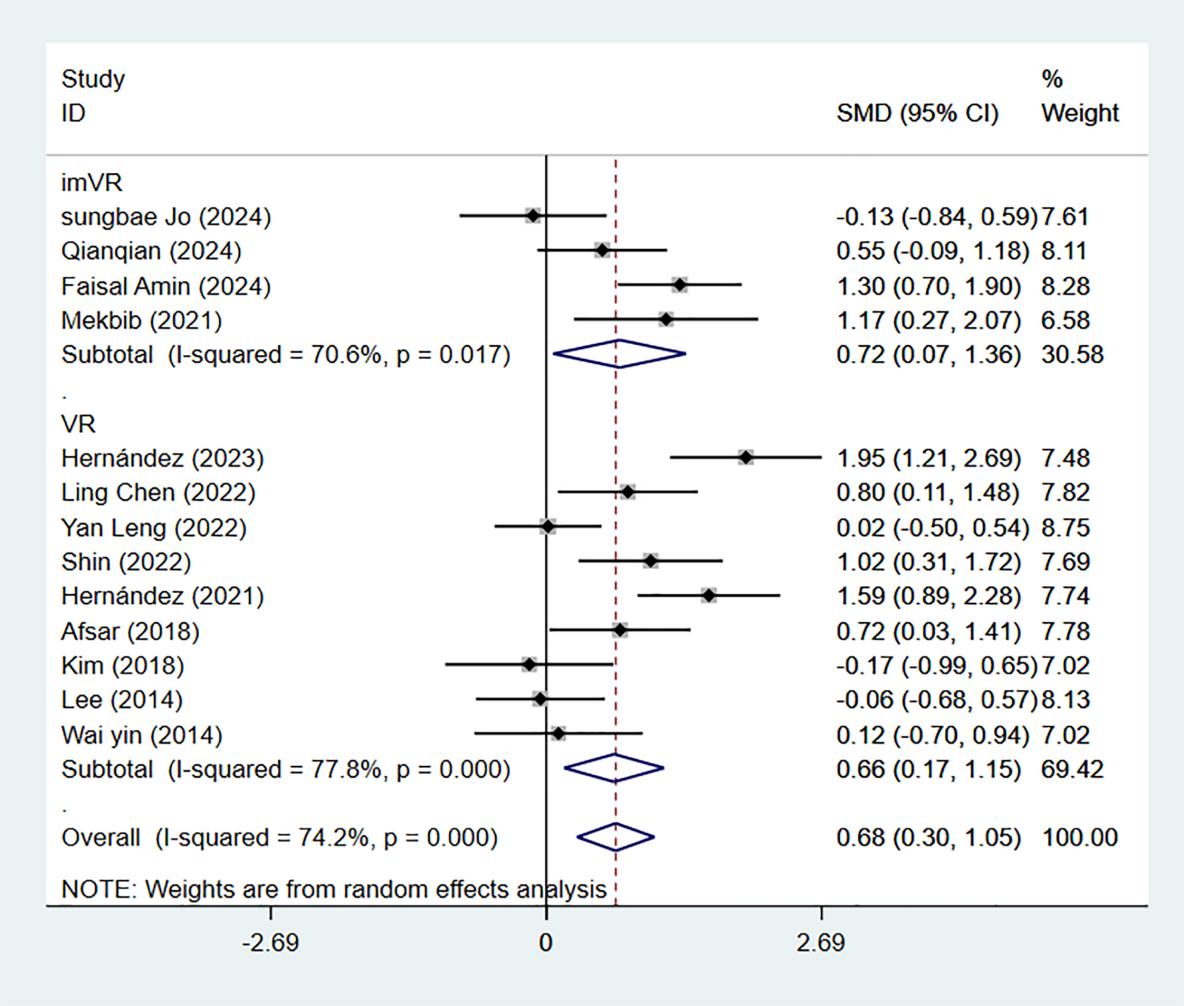
Forest plot of subgroup analysis of FMA-UE by intervention types.

Thirteen studies employed the FMA-UE scoring scale and were divided into two groups. Among these, four studies used immersive VR as the intervention method, and our meta-analysis yielded an SMD of 0.72 (95% CI [−0.07–1.36]; *P* = 0.030; *I*^2^ = 70.6%). The remaining nine studies utilized non-immersive VR, with an analysis result of SMD = 0.68 (95% CI [0.30–1.05]; *P* = 0.008; *I*^2^ = 77.8%). Both immersive and non-immersive VR training groups showed statistically significant differences compared to traditional rehabilitation (Fig. 3).

##### Subgroup analysis by intervention duration.

**Figure 4:**
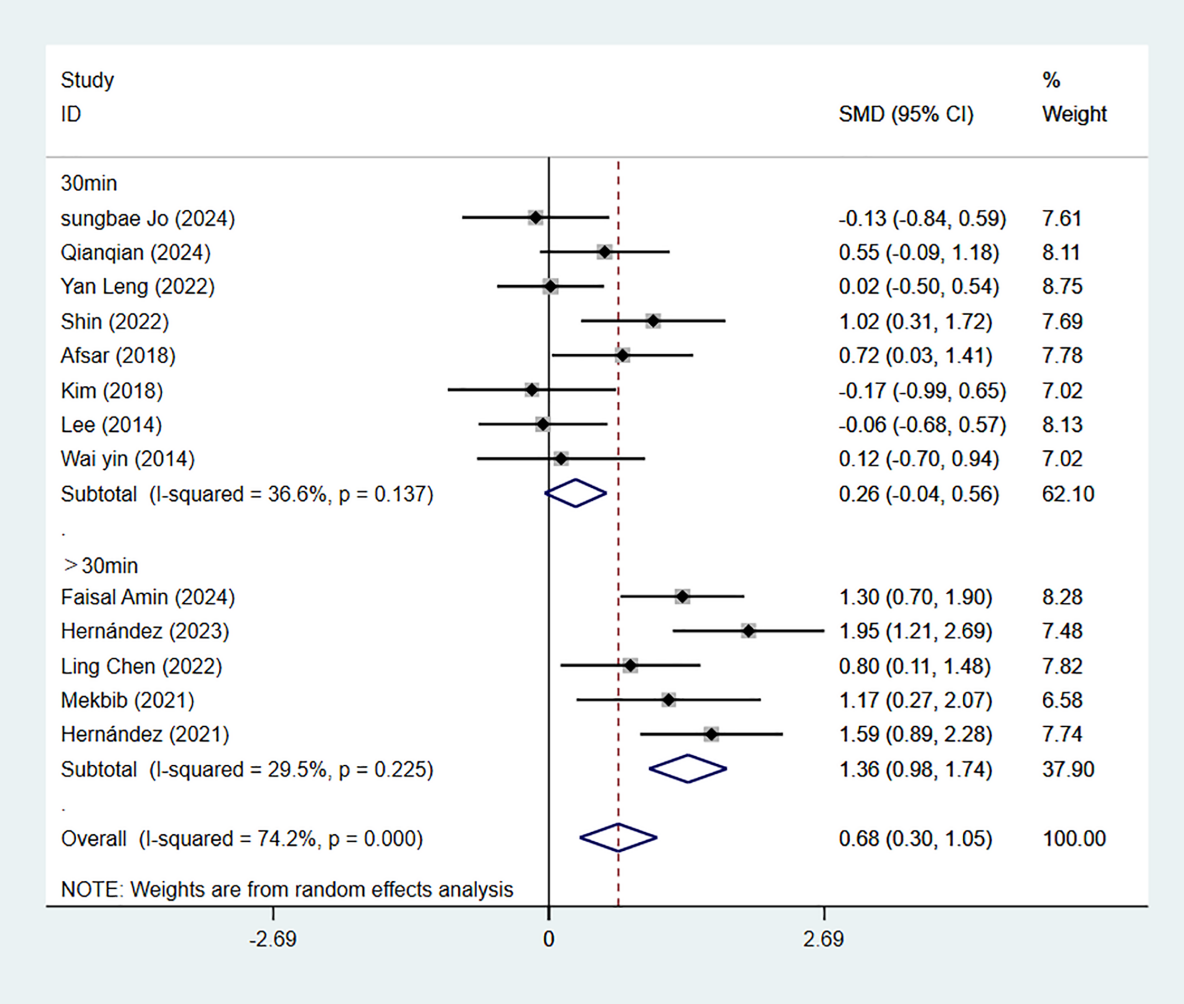
Forest plot of subgroup analysis of FMA-UE by intervention time.

Thirteen studies utilized the FMA-UE scoring scale, divided into two groups based on the intervention duration. Eight studies employed a 30-minute intervention, with an SMD of 0.26 (95% CI [−0.04–0.56]; *P* = 0.090, *I*^2^ = 36.6%). The remaining five studies with an intervention duration exceeding 30 min demonstrated superior outcomes for the VR training group compared to traditional rehabilitation, with a statistically significant difference (SMD = 1.36, 95% CI [0.98–1.94]; *P* = 0.000; *I*^2^ = 29.5%) (Fig. 4).

##### Subgroup analysis by intervention frequency.

**Figure 5:**
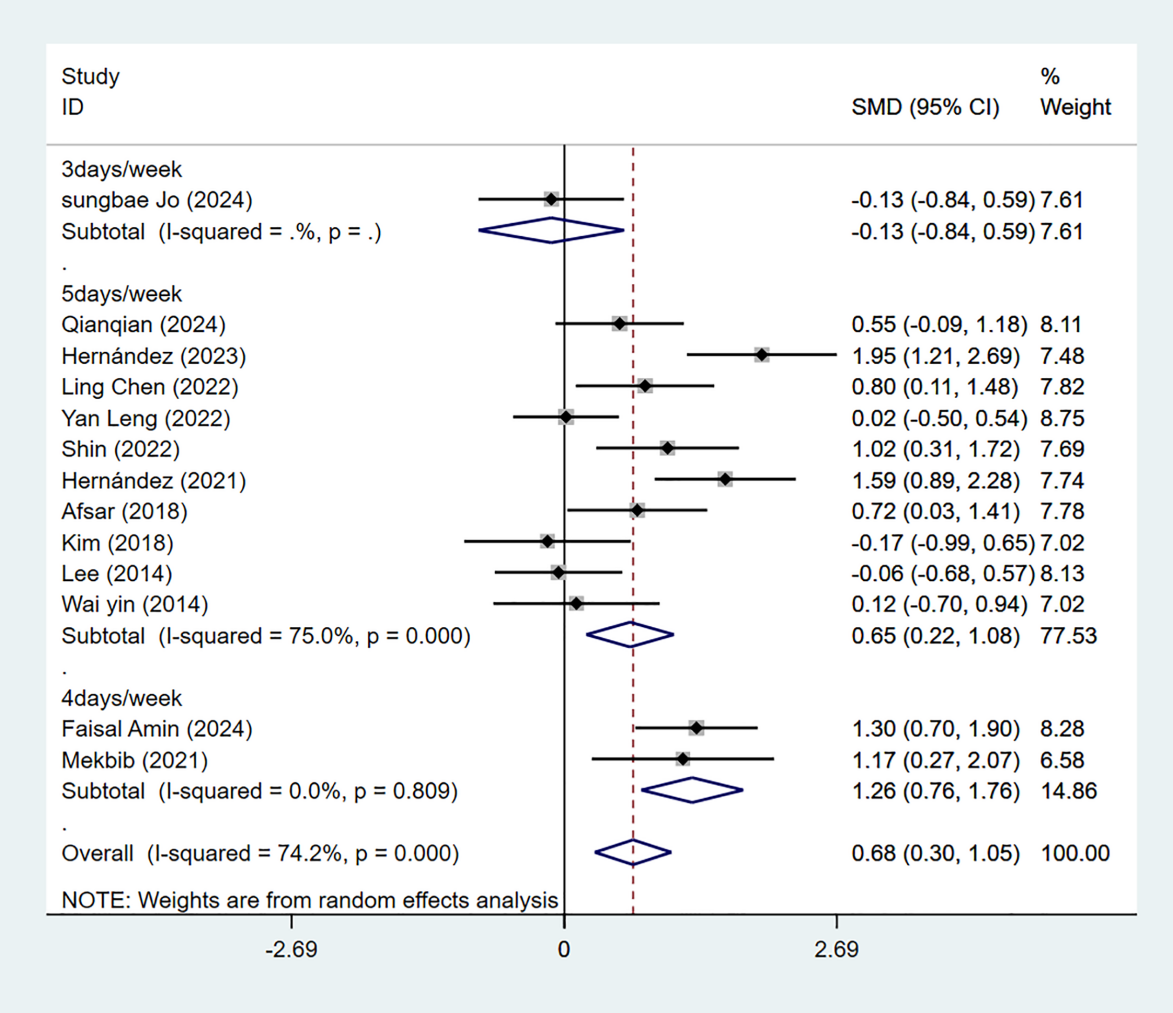
Forest plot of subgroup analysis of FMA-UE by the frequency of intervention.

Thirteen studies used the FMA-UE scoring scale, divided into three groups based on intervention frequency. One study employed a frequency of 3 days per week, with a meta-analysis result of SMD = −0.13 (95% CI [−0.84–0.59]; *P* = 0.726). Two studies employed 4 days per week, with an SMD of 1.26 (95% CI [0.76–1.76]; *P* = 0.000; *I*^2^ = 0.0%). Ten studies used 5 days per week, with an SMD = 0.65 (95% CI [0.22–1.08]; *P* = 0.003; *I*^2^ = 75.0%). Therefore, VR training with 4 or 5 days per week was superior to traditional rehabilitation, with statistically significant differences (Fig. 5).

##### Subgroup analysis by total intervention duration.

**Figure 6:**
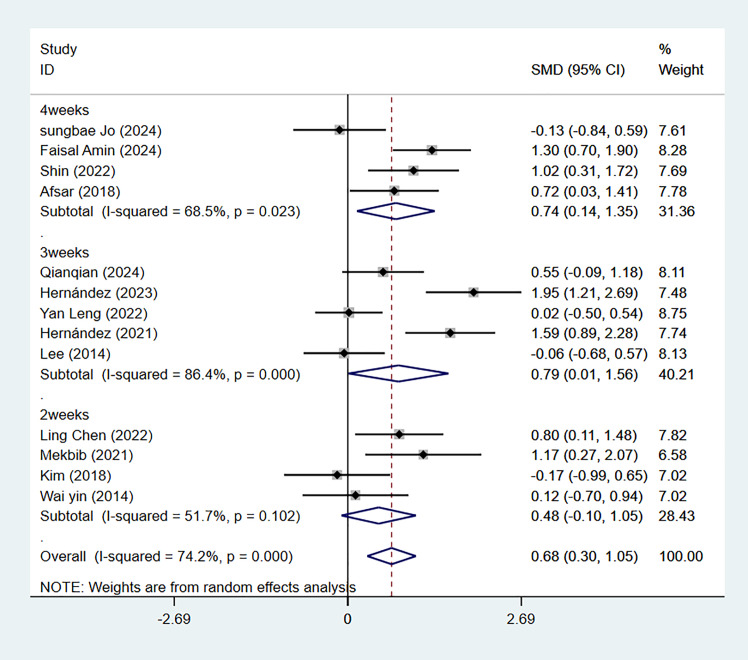
Forest plot of subgroup analysis of FMA-UE by total weeks of intervention.

Thirteen studies used the FMA-UE scoring scale, divided into three groups based on total intervention duration. Four studies had a total intervention duration of 2 weeks, with an SMD of 0.48 (95% CI [−0.10–1.05]; *P* = 0.103; *I*^2^ = 51.7%). Five studies had a total duration of 3 weeks, with an SMD of 0.79 (95% CI [0.01–1.56]; *P* = 0.046; *I*^2^ = 86.4%). Four studies had a total intervention duration of 4 weeks, yielding an SMD of 0.74 (95% CI [0.14–1.35]; *P* = 0.015; *I*^2^ = 68.5%). The meta-analysis indicated that VR training lasting 3 weeks or longer significantly outperformed traditional rehabilitation (*P* < 0.05)(Fig. 6).

##### Subgroup analysis by disease duration at inclusion.

Twelve studies assessed outcomes using the FMA-UE scale and were stratified into two groups according to the time since stroke at patient inclusion. In six studies, patients were enrolled within less than 6 months after stroke, yielding a SMD of 0.77 (95% CI [0.12–1.41]; *P* = 0.021; *I*^2^ = 82.8%). In the remaining six studies, patients were enrolled within less than 3 months after stroke, with an SMD of 0.47 (95% CI [0.01–0.93]; *P* = 0.044; *I*^2^ = 55.5%). The meta-analysis indicated that, irrespective of whether the disease duration was less than 3 months or less than 6 months, VR–based training was significantly more effective than conventional rehabilitation (*P* < 0.05) (Fig. S8).

#### Subgroup analysis of BBT

##### Subgroup analysis by intervention method.

Seven studies utilized the BBT rating scale and were divided into two groups. Five studies employed non-immersive VR as the intervention method, with an SMD of 0.16 (95% CI [−0.15–0.46]; *P* = 0.316; *I*^2^ = 18.1%). Two studies utilized immersive VR as the intervention method, and the meta-analysis results showed SMD = 1.08 (95% CI [0.20–1.97]; *P* = 0.016; *I*^2^ = 70.4%). The analysis indicated that immersive VR could better ameliorate BBT scores significantly than conventional rehabilitation, with statistically significant differences (Fig. S9).

##### Subgroup analysis by total intervention duration.

Seven studies used the BBT rating scale and were divided into two groups. Three studies had an intervention duration of ≤ 3 weeks, with a SMD = −0.03 (95% CI [−0.47–0.41]; *P* = 0.882; *I*^2^ = 0.0%). Four studies employed a total intervention duration of 4 weeks of VR training, with an SMD = 0.71 (95% CI [0.06–1.36]; *P* = 0.032; *I*^2^ = 80.0%). The BBT scores after 4 weeks were markedly different between VR training and conventional rehabilitation (Fig. S10). Furthermore, this study also implemented subgroup analyses based on different intervention frequencies (3/4/5 days/week) and intervention durations (30 min and >30 min). The VR training group exhibited no better BBT scores than the conventional rehabilitation cohort, and the differences were not statistically significant.

##### Subgroup analysis by disease duration at inclusion.

Six studies assessed outcomes using the BBT and were stratified into two subgroups according to disease duration. In two studies, patients were enrolled within less than 6 months after stroke, with an SMD of 0.30 (95% CI [−0.53–1.13]; *P* = 0.477; *I*^2^ = 64.2%). In four studies, patients were enrolled within less than three months after stroke, yielding an SMD of 0.07 (95% CI [−0.21–0.35]; *P* = 0.619; *I*^2^ = 0.0%). The meta-analysis indicated that, regardless of whether the disease duration was less than 3 months or less than 6 months, VR–based training was not significantly superior to conventional rehabilitation (*P* > 0.05) (Fig. S11).

#### Subgroup analysis of ARAT

##### Subgroup analysis by intervention method.

**Figure 7:**
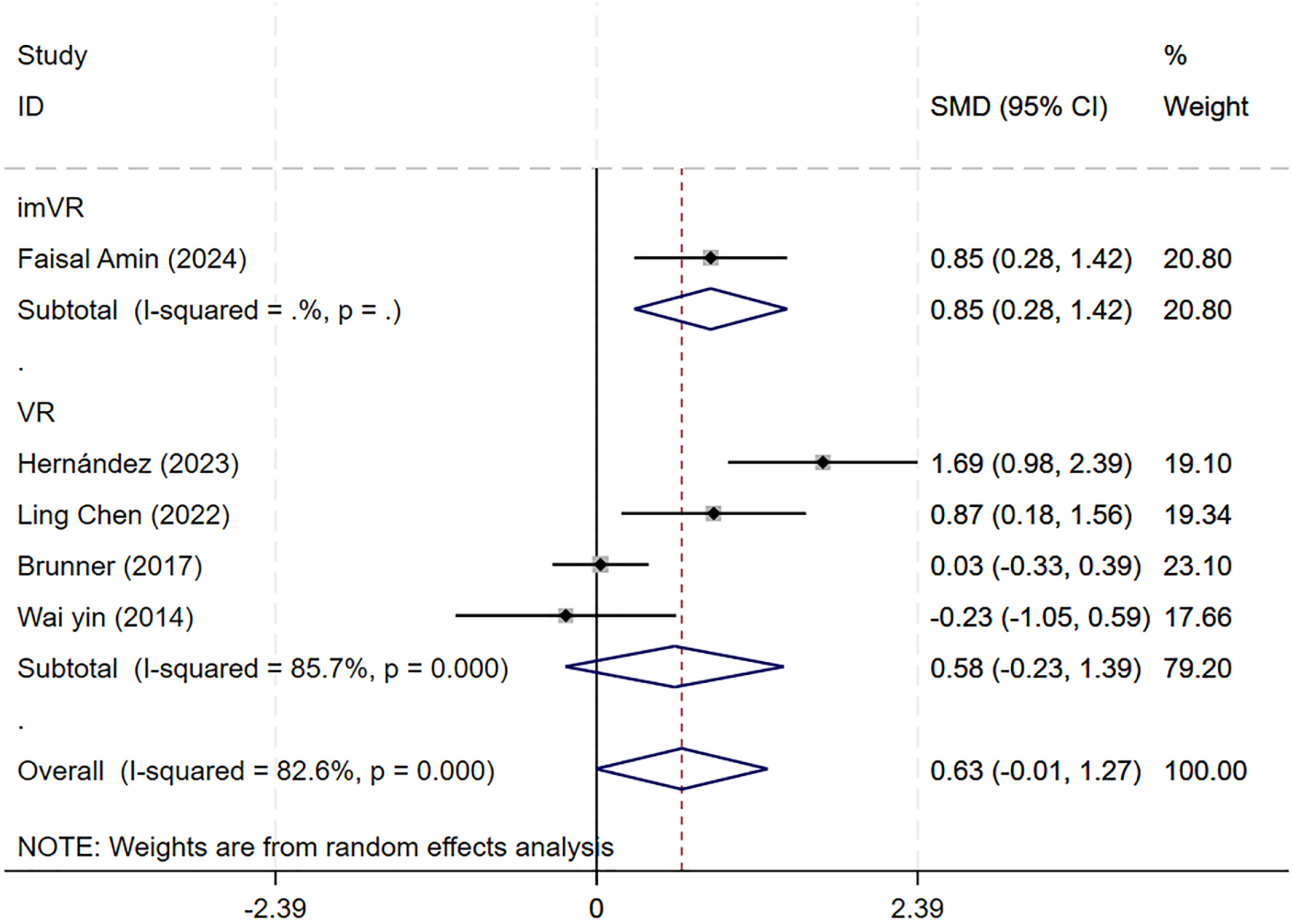
Forest plot of subgroup analysis of ARAT by intervention types.

Five studies used the ARAT scoring scale and were divided into two groups. Four studies employed non-immersive VR as the intervention method, and the SMD was 0.58 (95% CI [−0.23–1.39]; *P* = 0.162). One study utilized immersive VR as the intervention, with an SMD of 0.85 (95% CI [0.28–1.42]; *P* = 0.003). These findings indicated that the ARAT scores in the immersive VR training group significantly differed from those in the traditional rehabilitation group, with statistical significance (Fig. 7).

##### Subgroup analysis by intervention duration.

One study had an intervention duration of 30 min, with an SMD of −0.23 (95% CI [−1.05–0.59]; *P* = 0.584). Four studies had an intervention time of over 30 min, with an SMD of 0.82 (95% CI [0.10–1.54]; *P* = 0.026). A statistically significant difference in ARAT scores was noted between VR training with an intervention duration of greater than 30 min and traditional rehabilitation (Fig. S12). Furthermore, subgroup analysis was executed by different intervention frequencies (4 days/week and 5 days/week), intervention durations (2 weeks and >2 weeks). VR training failed to outperform traditional rehabilitation in terms of enhancing ARAT, with no statistically significant difference.

### Publication bias evaluation

A publication bias analysis was conducted on the 15 studies included. Funnel plots for FMA-UE, BBT, and ARAT were symmetrical. Egger’s test results were *P* = 0.535, *P* = 0.542, and *P* = 0.357, suggesting no publication bias in our study (Figs. S13–S15).

## Discussion

The analysis of 15 studies in the present review demonstrated that VR training significantly improved FMA-UE scores. In most studies, the experimental groups received VR combined with conventional rehabilitation, whereas some studies integrated VR with mirror therapy, real-world exposure therapy, or occupational therapy. Regardless of the rehabilitation strategy with which VR was combined, its therapeutic effects were superior to those of conventional rehabilitation alone, which is consistent with the findings of Mekbib et al. (2021).

Our results further indicated that VR was not significantly superior to conventional rehabilitation in improving BBT and ARAT scores in patients with stroke. This may be attributable to the typical recovery trajectory of the upper limb after stroke, which often progresses from gross motor function to fine and dexterous movements, as reflected by BBT and ARAT assessments. The preferential recovery of gross motor function may therefore have influenced outcomes on these measures. In addition, most of the included studies did not provide a detailed stratification of wrist and hand paralysis severity, which may represent another factor affecting BBT and ARAT results. Data from three follow-up studies included in this review showed no statistically significant differences between VR and conventional rehabilitation in BBT and ARAT scores immediately after intervention or at 1–3 months of follow-up; however, improvements were observed in both groups, suggesting that VR and conventional rehabilitation are comparably effective.

Subgroup analyses revealed that immersive VR was superior to non-immersive VR in improving BBT and ARAT scores, consistent with the conclusions of Fang et al. (2022). Our team and other researchers (Laver et al., 2017) have proposed several possible explanations. First, with respect to VR rehabilitation systems, early applications evolved from simple, generic games to task-oriented programs targeting gross motor skills, and subsequently to immersive systems specifically designed for fine hand motor rehabilitation. Moreover, sensory feedback in immersive VR has expanded from purely visual input to multimodal integration of visual, auditory, and haptic feedback. Second, regarding VR training tasks, early VR technologies primarily relied on exoskeletons, optical sensors, and data gloves, focusing on shoulder and elbow movements such as abduction, adduction, flexion, and extension. Because these sensors were unable to accurately detect hand movements, patients could not engage in game-based training involving finger flexion, extension, abduction, adduction, or grasping and pinching tasks. Limited precision in fine movements and reliance on handheld controllers also negatively affected user experience. In contrast, contemporary immersive VR systems are centered on hand-tracking technology, enabling accurate real-time capture of hand movements. Third, in terms of mechanisms, stroke-related neural injury can alter motor cortex excitability and spinal pathways, thereby impairing motor function (Sun & Zehr, 2019). Immersive VR may activate mirror neurons through repetitive upper limb practice, facilitating cortical reorganization and neuroplasticity (Wang et al., 2014). Finally, it should be noted that the present study yielded a “moderate risk of bias” rating based on the Cochrane 1.0 risk-of-bias assessment tool. Given that only four studies specifically investigated immersive VR, these findings should be interpreted with caution.

Exercise intensity significantly impacts stroke rehabilitation outcomes. This study on intervention type, duration, frequency, and total weeks found that VR sessions of at least 30 min, 4–5 times weekly for a minimum of three weeks, effectively ameliorate upper limb motor function in subacute hemiplegia patients. Supporting studies (Schuster-Amft et al., 2018; Zhang, Wong & Qin, 2023) show that 45-minute VR sessions, 3–5 days weekly, enhance ULF, aligning with our results. While VR may not address all rehabilitation aspects, it increases training intensity. Additional training aids functional recovery, as shown by Schneider et al. (2016) and Lohse, Lang & Boyd (2014) found that high-dose therapy outperforms low-dose therapy, though optimal VR intensity, frequency, and duration require further research. Although the subgroup and sensitivity analyses failed to identify the primary sources of significant statistical heterogeneity, factors such as sample size, racial differences, task difficulty, task specificity, and participant motivation may have contributed to the substantial heterogeneity among studies, which could in turn affect the generalizability of certain results.

## Study Limitations

This study has several limitations. First, most of the included studies were small-scale trials with relatively short intervention durations, and follow-up periods were largely limited to 1–3 months. The lack of long-term follow-up data restricts the ability to evaluate the sustained effects of the interventions. Second, evidence from unpublished studies, data related to treatment adherence, and reporting of publication bias were insufficient, and the heterogeneity of VR game types may have introduced additional bias. Moreover, common post-stroke attention deficits were not adequately addressed, which may have led to deviations between the observed outcomes and the true treatment effects.

## Conclusion

VR training is significantly more effective than conventional rehabilitation in improving ULF as measured by FMA-UE in patients with subacute stroke. Although no clear advantages were observed in BBT and ARAT scores, the overall post-intervention and follow-up outcomes indicate that VR combined with other rehabilitation approaches is comparable to conventional rehabilitation. Future studies should incorporate longer follow-up periods to assess the durability of VR-related benefits. In subsequent randomized controlled trials, stratification of wrist and hand function is recommended, with particular emphasis on evaluating the effects of VR on distal fine motor control and dexterity training.

##  Supplemental Information

10.7717/peerj.21073/supp-1 Supplemental Information 1 PRISMA checklist

10.7717/peerj.21073/supp-2 Supplemental Information 2 The intended audience

10.7717/peerj.21073/supp-3 Supplemental Information 3 Search strategy

10.7717/peerj.21073/supp-4 Supplemental Information 4 Risk of bias summary

10.7717/peerj.21073/supp-5 Supplemental Information 5 Risk of bias graph

10.7717/peerj.21073/supp-6 Supplemental Information 6 Sensitivity analysis of FMA-UE

10.7717/peerj.21073/supp-7 Supplemental Information 7 Forest plot of BBT

10.7717/peerj.21073/supp-8 Supplemental Information 8 Sensitivity analysis of BBT

10.7717/peerj.21073/supp-9 Supplemental Information 9 Forest plot of ARAT

10.7717/peerj.21073/supp-10 Supplemental Information 10 Sensitivity analysis of ARAT

10.7717/peerj.21073/supp-11 Supplemental Information 11 Forest plot showing subgroup analysis of FMA-UE by disease duration at inclusion

10.7717/peerj.21073/supp-12 Supplemental Information 12 Forest plot of subgroup analysis of BBT by intervention types

10.7717/peerj.21073/supp-13 Supplemental Information 13 Forest plot of subgroup analysis of BBT total weeks of intervention

10.7717/peerj.21073/supp-14 Supplemental Information 14 Forest plot showing subgroup analysis of BBT by disease duration at inclusion

10.7717/peerj.21073/supp-15 Supplemental Information 15 Forest plot of subgroup analysis of ARAT by intervention time

10.7717/peerj.21073/supp-16 Supplemental Information 16 Funnel plot of FMA-UE

10.7717/peerj.21073/supp-17 Supplemental Information 17 Funnel plot of BBT

10.7717/peerj.21073/supp-18 Supplemental Information 18 Funnel plot of ARAT

## Additional Information and Declarations

### Data Availability

The following information was supplied regarding data availability: This is a Systematic Review/Meta-analysis.
