# Supplementary material for: The influence of virtual reality technology on upper limb motor function in subacute stroke: a systematic review and meta-analysis
Source: PeerJ. 2026 Apr 16;14:e21073. doi: 10.7717/peerj.21073 (PMC13092230; doi:10.7717/peerj.21073)
Supplement: Supplemental Information 2 [file peerj-14-21073-s002.docx]

**The audience it is intended for：**

1. Medical professionals: Personnel involved in the treatment and rehabilitation process of stroke patients include rehabilitation physicians, neurologists, physical therapists, etc.

2. Researchers: Researchers in the fields of medical engineering and rehabilitation medicine who focus on the rehabilitation mechanism, optimization, and improvement of virtual reality technology in stroke rehabilitation.

3. Healthcare managers or policymakers: Those who pay attention to the impact of virtual reality technology on the allocation and application of medical resources, so as to formulate relevant policies and measures.
