# Supplementary material for: The influence of virtual reality technology on upper limb motor function in subacute stroke: a systematic review and meta-analysis
Source: PeerJ. 2026 Apr 16;14:e21073. doi: 10.7717/peerj.21073 (PMC13092230; doi:10.7717/peerj.21073)
Supplement: Supplemental Information 3 [file peerj-14-21073-s003.docx]

**Table S1 Search strategy**

**Cochrane**

#1 MeSH descriptor: [Virtual Reality] explode all trees

#2 (virtual reality):ti,ab,kw OR (Reality, Virtual):ti,ab,kw OR (Virtual Reality, Educational):ti,ab,kw OR (Educational Virtual Realities):ti,ab,kw OR (Educational Virtual Reality):ti,ab,kw

#3 (Reality, Educational Virtual):ti,ab,kw OR (Virtual Realities, Educational):ti,ab,kw OR (Virtual Reality, Instructional):ti,ab,kw OR (Instructional Virtual Realities):ti,ab,kw OR (Instructional Virtual Reality):ti,ab,kw

#4 (Realities, Instructional Virtual):ti,ab,kw AND (Reality, Instructional Virtual):ti,ab,kw AND (Virtual Realities, Instructional):ti,ab,kw

#5 #1 or #2 or #3 or #4

#6 MeSH descriptor: [Stroke] explode all trees

#7 (stroke):ti,ab,kw OR (Strokes):ti,ab,kw OR (Cerebrovascular Accident):ti,ab,kw OR (Cerebrovascular Accidents):ti,ab,kw OR (Cerebral Stroke):ti,ab,kw

#8 (Cerebral Strokes):ti,ab,kw OR (Stroke, Cerebral):ti,ab,kw OR (Strokes, Cerebral):ti,ab,kw OR (Cerebrovascular Apoplexy):ti,ab,kw OR (Apoplexy, Cerebrovascular):ti,ab,kw

#9 (Vascular Accident, Brain):ti,ab,kw OR (Brain Vascular Accident):ti,ab,kw OR (Brain Vascular Accidents):ti,ab,kw OR (Vascular Accidents, Brain):ti,ab,kw OR (Cerebrovascular Stroke):ti,ab,kw

#10 (Cerebrovascular Strokes):ti,ab,kw OR (Stroke, Cerebrovascular):ti,ab,kw OR (Strokes, Cerebrovascular):ti,ab,kw OR (Apoplexy):ti,ab,kw OR (CVA (Cerebrovascular Accident)):ti,ab,kw

#11 (CVAs (Cerebrovascular Accident)):ti,ab,kw OR (Stroke, Acute):ti,ab,kw OR (Acute Stroke):ti,ab,kw OR (Acute Strokes):ti,ab,kw OR (Strokes, Acute):ti,ab,kw

#12 (Cerebrovascular Accident, Acute):ti,ab,kw OR (Acute Cerebrovascular Accident):ti,ab,kw OR (Acute Cerebrovascular Accidents):ti,ab,kw OR (Cerebrovascular Accidents, Acute):ti,ab,kw

#13 #6 or #7 or #8 or #9 or #10 or #11 or #12

#14 #5 and #13

Result 909

**Pubmed**

#1 virtual reality[MeSH Terms]

#2((((((((((((virtual reality[Title/Abstract]) OR (Reality, Virtual[Title/Abstract])) OR (Virtual Reality, Educational[Title/Abstract])) OR (Educational Virtual Realities[Title/Abstract])) OR (Educational Virtual Reality[Title/Abstract])) OR (Reality, Educational Virtual[Title/Abstract])) OR (Virtual Realities, Educational[Title/Abstract])) OR (Virtual Reality, Instructional[Title/Abstract])) OR (Instructional Virtual Realities[Title/Abstract])) OR (Instructional Virtual Reality[Title/Abstract])) OR (Realities, Instructional Virtual[Title/Abstract])) OR (Reality, Instructional Virtual[Title/Abstract])) OR (Virtual Realities, Instructional[Title/Abstract])

#3 (virtual reality[MeSH Terms]) OR (((((((((((((virtual reality[Title/Abstract]) OR (Reality, Virtual[Title/Abstract])) OR (Virtual Reality, Educational[Title/Abstract])) OR (Educational Virtual Realities[Title/Abstract])) OR (Educational Virtual Reality[Title/Abstract])) OR (Reality, Educational Virtual[Title/Abstract])) OR (Virtual Realities, Educational[Title/Abstract])) OR (Virtual Reality, Instructional[Title/Abstract])) OR (Instructional Virtual Realities[Title/Abstract])) OR (Instructional Virtual Reality[Title/Abstract])) OR (Realities, Instructional Virtual[Title/Abstract])) OR (Reality, Instructional Virtual[Title/Abstract])) OR (Virtual Realities, Instructional[Title/Abstract]))

#4 Stroke[MeSH Terms]

#5 ((((((((((((((((((((((((((((Stroke[Title/Abstract]) OR (Strokes[Title/Abstract])) OR (Cerebrovascular Accident[Title/Abstract])) OR (Cerebrovascular Accidents[Title/Abstract])) OR (Cerebral Stroke[Title/Abstract])) OR (Cerebral Strokes[Title/Abstract])) OR (Stroke, Cerebral[Title/Abstract])) OR (Strokes, Cerebral[Title/Abstract])) OR (Cerebrovascular Apoplexy[Title/Abstract])) OR (Apoplexy, Cerebrovascular[Title/Abstract])) OR (Vascular Accident, Brain[Title/Abstract])) OR (Brain Vascular Accident[Title/Abstract])) OR (Brain Vascular Accidents[Title/Abstract])) OR (Vascular Accidents, Brain[Title/Abstract])) OR (Cerebrovascular Stroke[Title/Abstract])) OR (Cerebrovascular Strokes[Title/Abstract])) OR (Stroke, Cerebrovascular[Title/Abstract])) OR (Strokes, Cerebrovascular[Title/Abstract])) OR (Apoplexy[Title/Abstract])) OR (CVA (Cerebrovascular Accident[Title/Abstract]))) OR (CVAs (Cerebrovascular Accident[Title/Abstract]))) OR (Stroke, Acute[Title/Abstract])) OR (Acute Stroke[Title/Abstract])) OR (Acute Strokes[Title/Abstract])) OR (Strokes, Acute[Title/Abstract])) OR (Cerebrovascular Accident, Acute[Title/Abstract])) OR (Acute Cerebrovascular Accident[Title/Abstract])) OR (Acute Cerebrovascular Accidents[Title/Abstract])) OR (Cerebrovascular Accidents, Acute[Title/Abstract])

#6 (Stroke[MeSH Terms]) OR (((((((((((((((((((((((((((((Stroke[Title/Abstract]) OR (Strokes[Title/Abstract])) OR (Cerebrovascular Accident[Title/Abstract])) OR (Cerebrovascular Accidents[Title/Abstract])) OR (Cerebral Stroke[Title/Abstract])) OR (Cerebral Strokes[Title/Abstract])) OR (Stroke, Cerebral[Title/Abstract])) OR (Strokes, Cerebral[Title/Abstract])) OR (Cerebrovascular Apoplexy[Title/Abstract])) OR (Apoplexy, Cerebrovascular[Title/Abstract])) OR (Vascular Accident, Brain[Title/Abstract])) OR (Brain Vascular Accident[Title/Abstract])) OR (Brain Vascular Accidents[Title/Abstract])) OR (Vascular Accidents, Brain[Title/Abstract])) OR (Cerebrovascular Stroke[Title/Abstract])) OR (Cerebrovascular Strokes[Title/Abstract])) OR (Stroke, Cerebrovascular[Title/Abstract])) OR (Strokes, Cerebrovascular[Title/Abstract])) OR (Apoplexy[Title/Abstract])) OR (CVA (Cerebrovascular Accident[Title/Abstract]))) OR (CVAs (Cerebrovascular Accident[Title/Abstract]))) OR (Stroke, Acute[Title/Abstract])) OR (Acute Stroke[Title/Abstract])) OR (Acute Strokes[Title/Abstract])) OR (Strokes, Acute[Title/Abstract])) OR (Cerebrovascular Accident, Acute[Title/Abstract])) OR (Acute Cerebrovascular Accident[Title/Abstract])) OR (Acute Cerebrovascular Accidents[Title/Abstract])) OR (Cerebrovascular Accidents, Acute[Title/Abstract]))

#7 ((virtual reality[MeSH Terms]) OR (((((((((((((virtual reality[Title/Abstract]) OR (Reality, Virtual[Title/Abstract])) OR (Virtual Reality, Educational[Title/Abstract])) OR (Educational Virtual Realities[Title/Abstract])) OR (Educational Virtual Reality[Title/Abstract])) OR (Reality, Educational Virtual[Title/Abstract])) OR (Virtual Realities, Educational[Title/Abstract])) OR (Virtual Reality, Instructional[Title/Abstract])) OR (Instructional Virtual Realities[Title/Abstract])) OR (Instructional Virtual Reality[Title/Abstract])) OR (Realities, Instructional Virtual[Title/Abstract])) OR (Reality, Instructional Virtual[Title/Abstract])) OR (Virtual Realities, Instructional[Title/Abstract]))) AND ((Stroke[MeSH Terms]) OR (((((((((((((((((((((((((((((Stroke[Title/Abstract]) OR (Strokes[Title/Abstract])) OR (Cerebrovascular Accident[Title/Abstract])) OR (Cerebrovascular Accidents[Title/Abstract])) OR (Cerebral Stroke[Title/Abstract])) OR (Cerebral Strokes[Title/Abstract])) OR (Stroke, Cerebral[Title/Abstract])) OR (Strokes, Cerebral[Title/Abstract])) OR (Cerebrovascular Apoplexy[Title/Abstract])) OR (Apoplexy, Cerebrovascular[Title/Abstract])) OR (Vascular Accident, Brain[Title/Abstract])) OR (Brain Vascular Accident[Title/Abstract])) OR (Brain Vascular Accidents[Title/Abstract])) OR (Vascular Accidents, Brain[Title/Abstract])) OR (Cerebrovascular Stroke[Title/Abstract])) OR (Cerebrovascular Strokes[Title/Abstract])) OR (Stroke, Cerebrovascular[Title/Abstract])) OR (Strokes, Cerebrovascular[Title/Abstract])) OR (Apoplexy[Title/Abstract])) OR (CVA (Cerebrovascular Accident[Title/Abstract]))) OR (CVAs (Cerebrovascular Accident[Title/Abstract]))) OR (Stroke, Acute[Title/Abstract])) OR (Acute Stroke[Title/Abstract])) OR (Acute Strokes[Title/Abstract])) OR (Strokes, Acute[Title/Abstract])) OR (Cerebrovascular Accident, Acute[Title/Abstract])) OR (Acute Cerebrovascular Accident[Title/Abstract])) OR (Acute Cerebrovascular Accidents[Title/Abstract])) OR (Cerebrovascular Accidents, Acute[Title/Abstract])))

Result 1529

**Embase**

#1 'virtual reality'/exp

#2 'virtual reality':ab,ti

#3'reality, virtual':ab,ti

#4'virtual reality, educational':ab,ti

#5'educational virtual realities':ab,ti

#6'educational virtual reality':ab,ti

#7'reality, educational virtual':ab,ti

#8'virtual realities, educational':ab,ti

#9'virtual reality, instructional':ab,ti

#10'instructional virtual realities':ab,ti

#11 'instructional virtual reality':ab,ti

#12 'realities, instructional virtual':ab,ti

#13 'reality, instructional virtual':ab,ti

#14 'virtual realities, instructional':ab,ti

#15 #1 OR #2 OR #3 OR #4 OR #5 OR #6 OR #7

OR #8 OR #9 OR #10 OR #11 OR #12 OR #13 OR #14

#16'cerebrovascular accident'/exp

#17'stroke':ab,ti

#18'strokes':ab,ti

#19'cerebrovascular accident':ab,ti

#20'cerebrovascular accidents':ab,ti

#21'cerebral stroke':ab,ti

#22'cerebral strokes':ab,ti

#23'stroke, cerebral':ab,ti

#24'strokes, cerebral':ab,ti

#25'cerebrovascular apoplexy':ab,ti

#26'apoplexy, cerebrovascular':ab,ti

#27'vascular accident, brain':ab,ti

#28'brain vascular accident':ab,ti

#29'brain vascular accidents':ab,ti

#30'vascular accidents, brain':ab,ti

#31'cerebrovascular stroke':ab,ti

#32'cerebrovascular strokes':ab,ti

#33'stroke, cerebrovascular':ab,ti

#34'strokes, cerebrovascular':ab,ti

#35'apoplexy':ab,ti

#36'cva (cerebrovascular accident)':ab,ti

#37'cvas (cerebrovascular accident)':ab,ti

#38'stroke, acute':ab,ti

#39'acute stroke':ab,ti

#40'acute strokes':ab,ti

#41'strokes, acute':ab,ti

#42'cerebrovascular accident, acute':ab,ti

#43'acute cerebrovascular accident':ab,ti

#44'acute cerebrovascular accidents':ab,ti

#45'cerebrovascular accidents, acute':ab,ti

#46#16 OR #17 OR #18 OR #19 OR #20 OR #21 OR #22 OR #23 OR #24 OR #25 OR #26 OR #27 OR #28 OR #29 OR #30 OR #31 OR #32 OR #33 OR #34 OR #35 OR #36 OR #37 OR #38 OR #39 OR #40 OR #41 OR #42 OR #43 OR #44 OR #45

#47#15 AND #46

Result:2010

**Web**

#1TS=(virtual reality) OR TS=(Reality, Virtual) OR TS=(Virtual Reality, Educational) OR TS=(Educational Virtual Realities) OR TS=(Educational Virtual Reality) OR TS=(Reality, Educational Virtual) OR TS=(Virtual Realities, Educational) OR TS=(Virtual Reality, Instructional) OR TS=(Instructional Virtual Realities) OR TS=(Instructional Virtual Reality) OR TS=(Realities, Instructional Virtual) OR TS=(Reality, Instructional Virtual) OR TS=(Virtual Realities, Instruction

TS=(Stroke) OR TS=(Strokes) OR TS=(Cerebrovascular Accident) OR TS=(Cerebrovascular Accidents) OR TS=(Cerebral Stroke) OR TS=(Cerebral Strokes) OR TS=(Stroke, Cerebral) OR TS=(Strokes, Cerebral) OR TS=(Cerebrovascular Apoplexy) OR TS=(Apoplexy, Cerebrovascular) OR TS=(Vascular Accident, Brain) OR TS=(Brain Vascular Accident) OR TS=(Brain Vascular Accidents) OR TS=(Vascular Accidents, Brain) OR TS=(Cerebrovascular Stroke) OR TS=(Cerebrovascular Strokes) OR TS=(Stroke, Cerebrovascular) OR TS=(Strokes, Cerebrovascular) OR TS=(Apoplexy) OR TS=(CVA (Cerebrovascular Accident)) OR TS=(CVAs (Cerebrovascular Accident)) OR TS=(Stroke, Acute) OR TS=(Acute Stroke) OR TS=(Acute Strokes) OR TS=(Strokes, Acute) OR TS=(Cerebrovascular Accident, Acute) OR TS=(Acute Cerebrovascular Accident) OR TS=(Acute Cerebrovascular Accidents) OR TS=(Cerebrovascular Accidents, Acute)

#2 TS=(Stroke) OR TS=(Strokes) OR TS=(Cerebrovascular Accident) OR TS=(Cerebrovascular Accidents) OR TS=(Cerebral Stroke) OR TS=(Cerebral Strokes) OR TS=(Stroke, Cerebral) OR TS=(Strokes, Cerebral) OR TS=(Cerebrovascular Apoplexy) OR TS=(Apoplexy, Cerebrovascular) OR TS=(Vascular Accident, Brain) OR TS=(Brain Vascular Accident) OR TS=(Brain Vascular Accidents) OR TS=(Vascular Accidents, Brain) OR TS=(Cerebrovascular Stroke) OR TS=(Cerebrovascular Strokes) OR TS=(Stroke, Cerebrovascular) OR TS=(Strokes, Cerebrovascular) OR TS=(Apoplexy) OR TS=(CVA (Cerebrovascular Accident)) OR TS=(CVAs (Cerebrovascular Accident)) OR TS=(Stroke, Acute) OR TS=(Acute Stroke) OR TS=(Acute Strokes) OR TS=(Strokes, Acute) OR TS=(Cerebrovascular Accident, Acute) OR TS=(Acute Cerebrovascular Accident) OR TS=(Acute Cerebrovascular Accidents) OR TS=(Cerebrovascular Accidents, Acute)

#3 #2 AND #1

Reuslt 3265
