# Supplementary figures and images for: The influence of virtual reality technology on upper limb motor function in subacute stroke: a systematic review and meta-analysis
Source: PeerJ. 2026 Apr 16;14:e21073. doi: 10.7717/peerj.21073 (PMC13092230; doi:10.7717/peerj.21073)

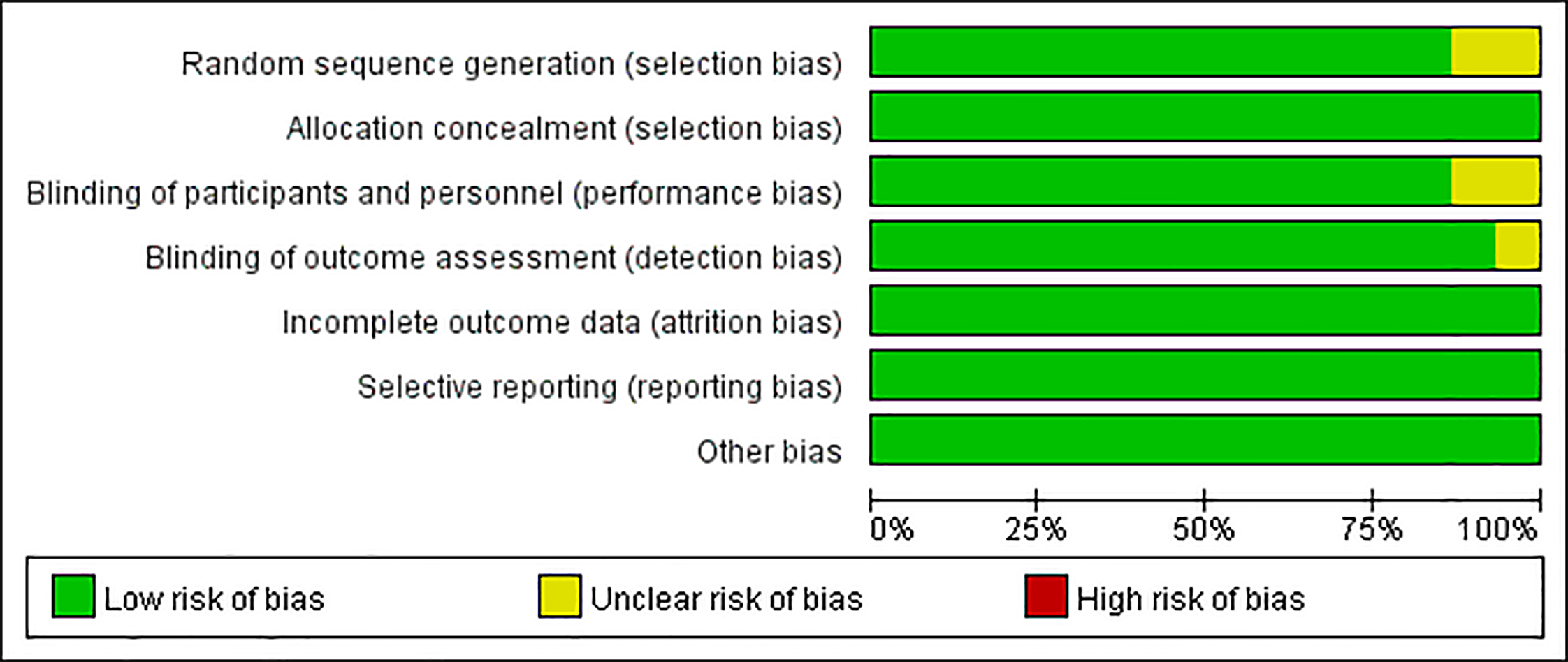

Supplement: Supplemental Information 4 [file peerj-14-21073-s004.png]

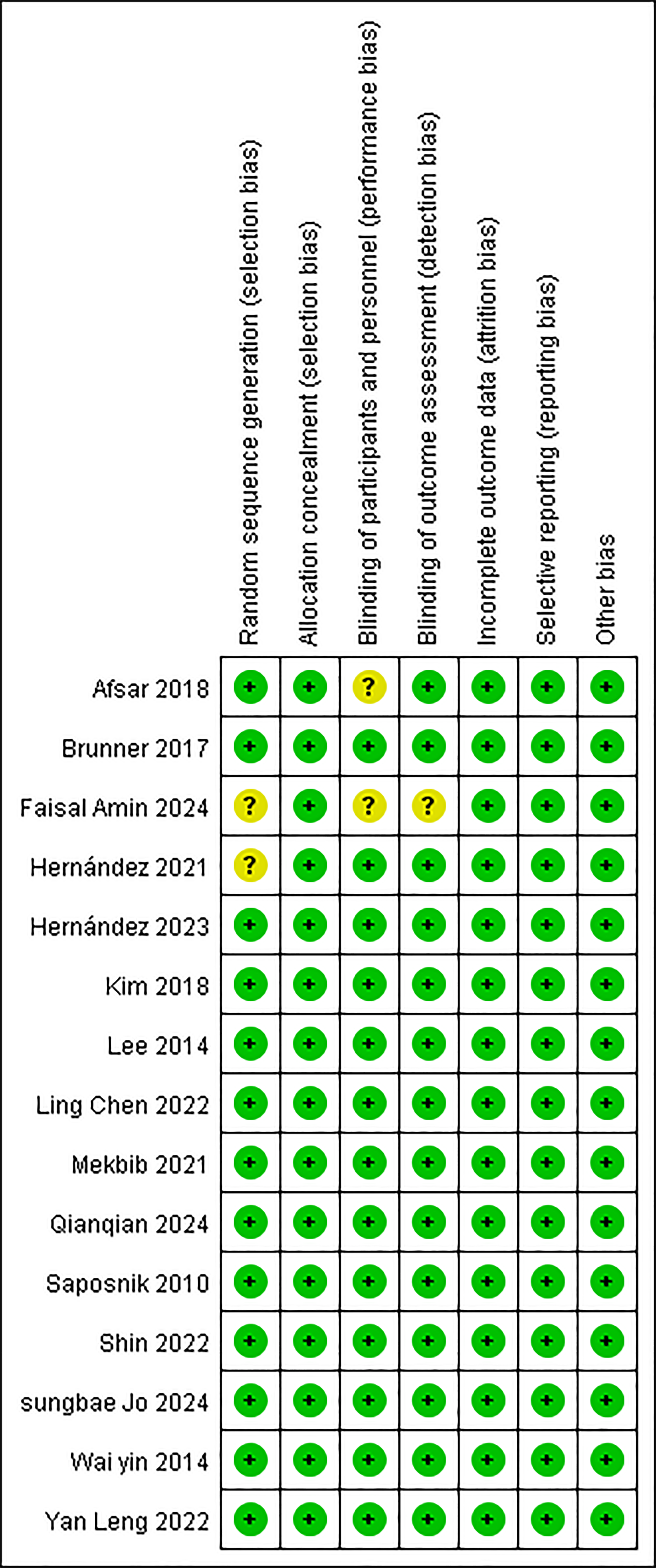

Supplement: Supplemental Information 5 [file peerj-14-21073-s005.png]

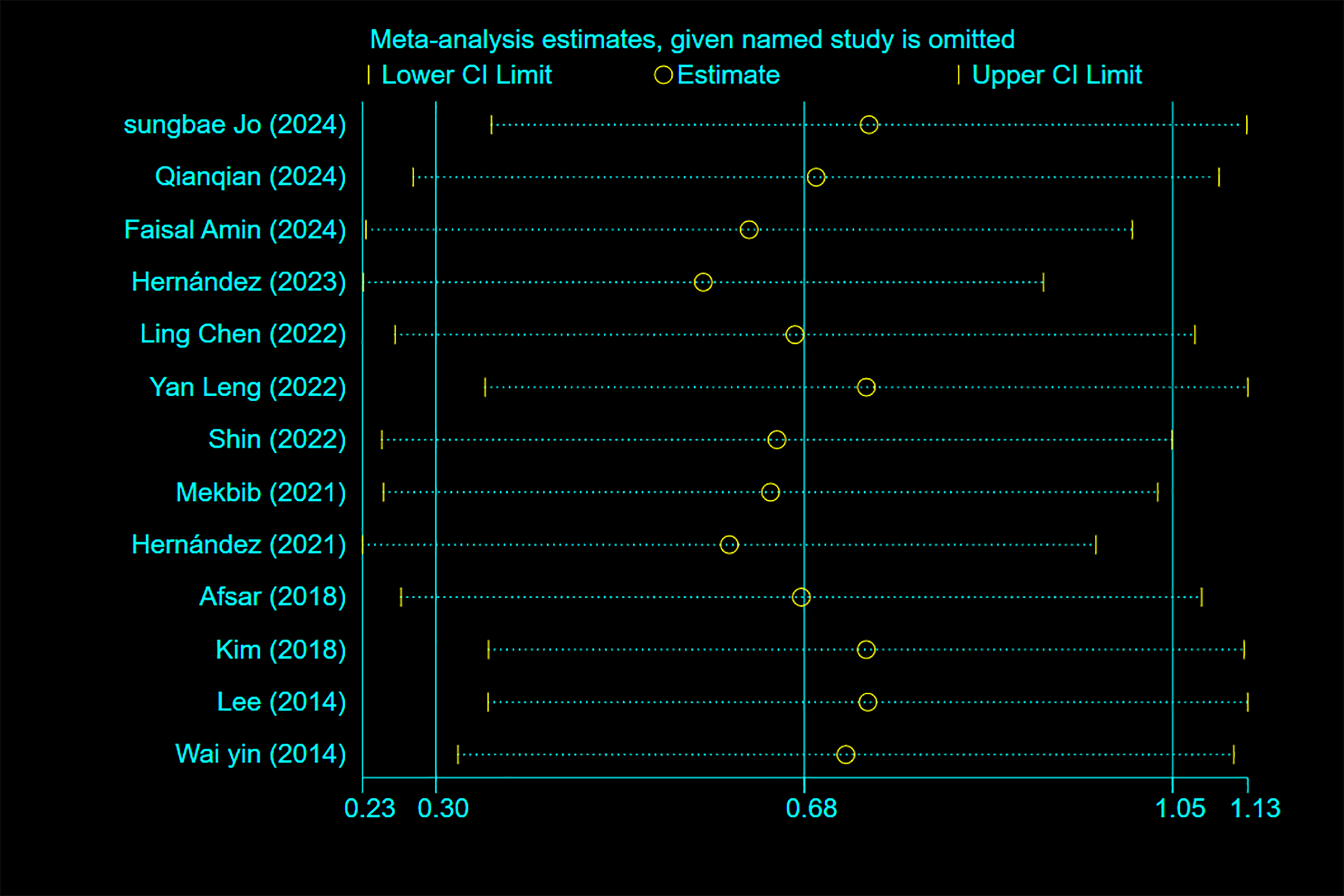

Supplement: Supplemental Information 6 [file peerj-14-21073-s006.png]

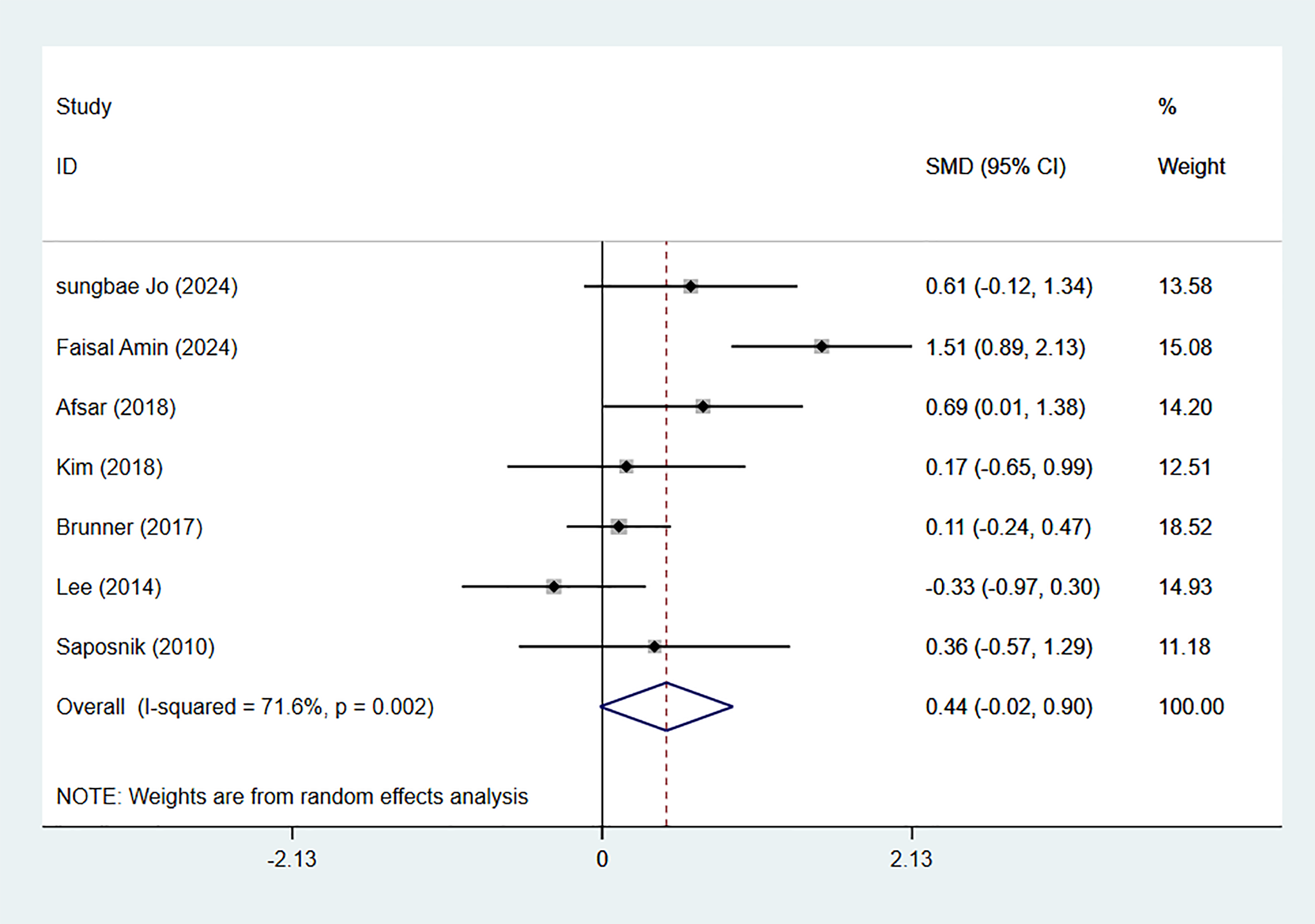

Supplement: Supplemental Information 7 [file peerj-14-21073-s007.png]

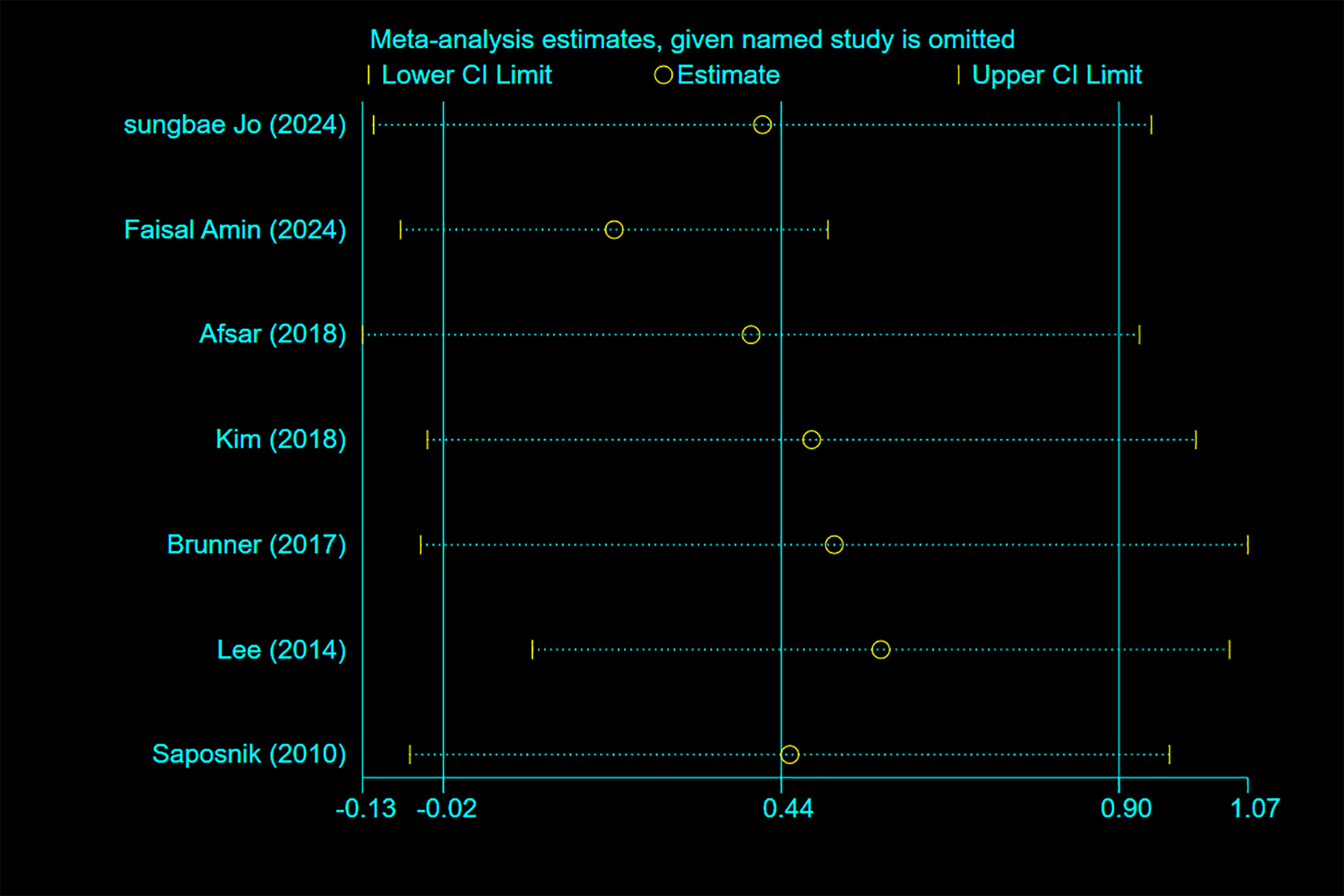

Supplement: Supplemental Information 8 [file peerj-14-21073-s008.png]

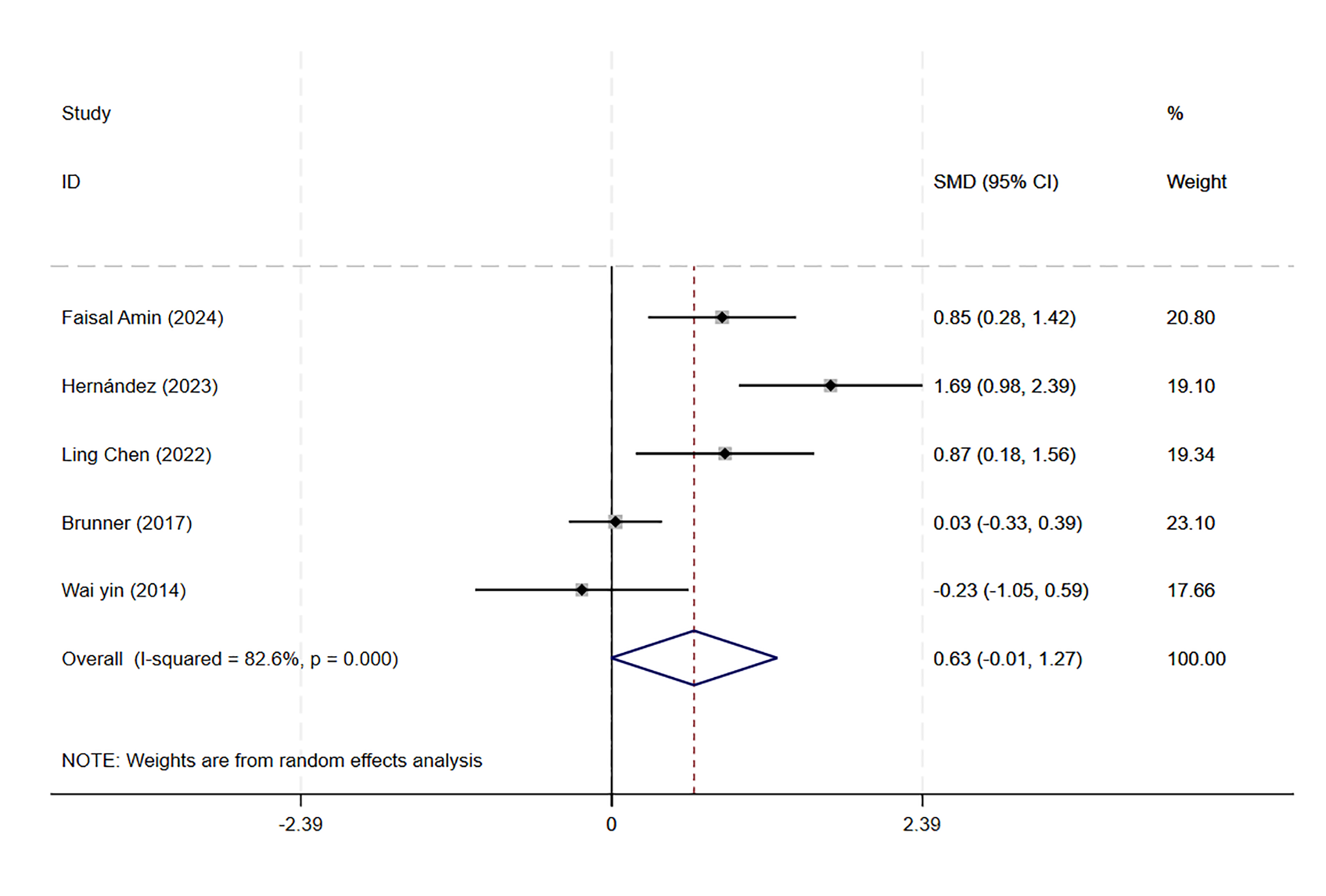

Supplement: Supplemental Information 9 [file peerj-14-21073-s009.png]

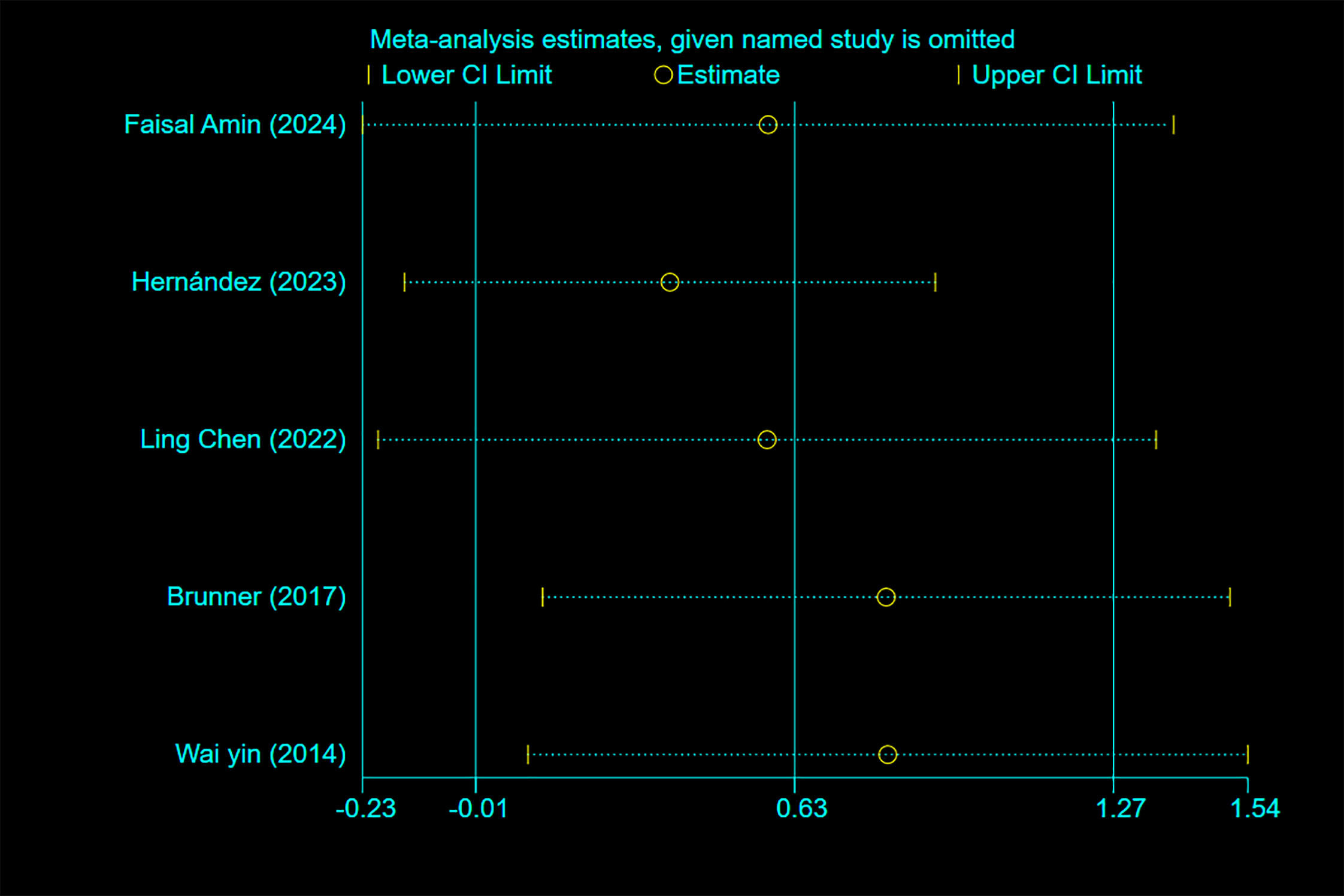

Supplement: Supplemental Information 10 [file peerj-14-21073-s010.png]

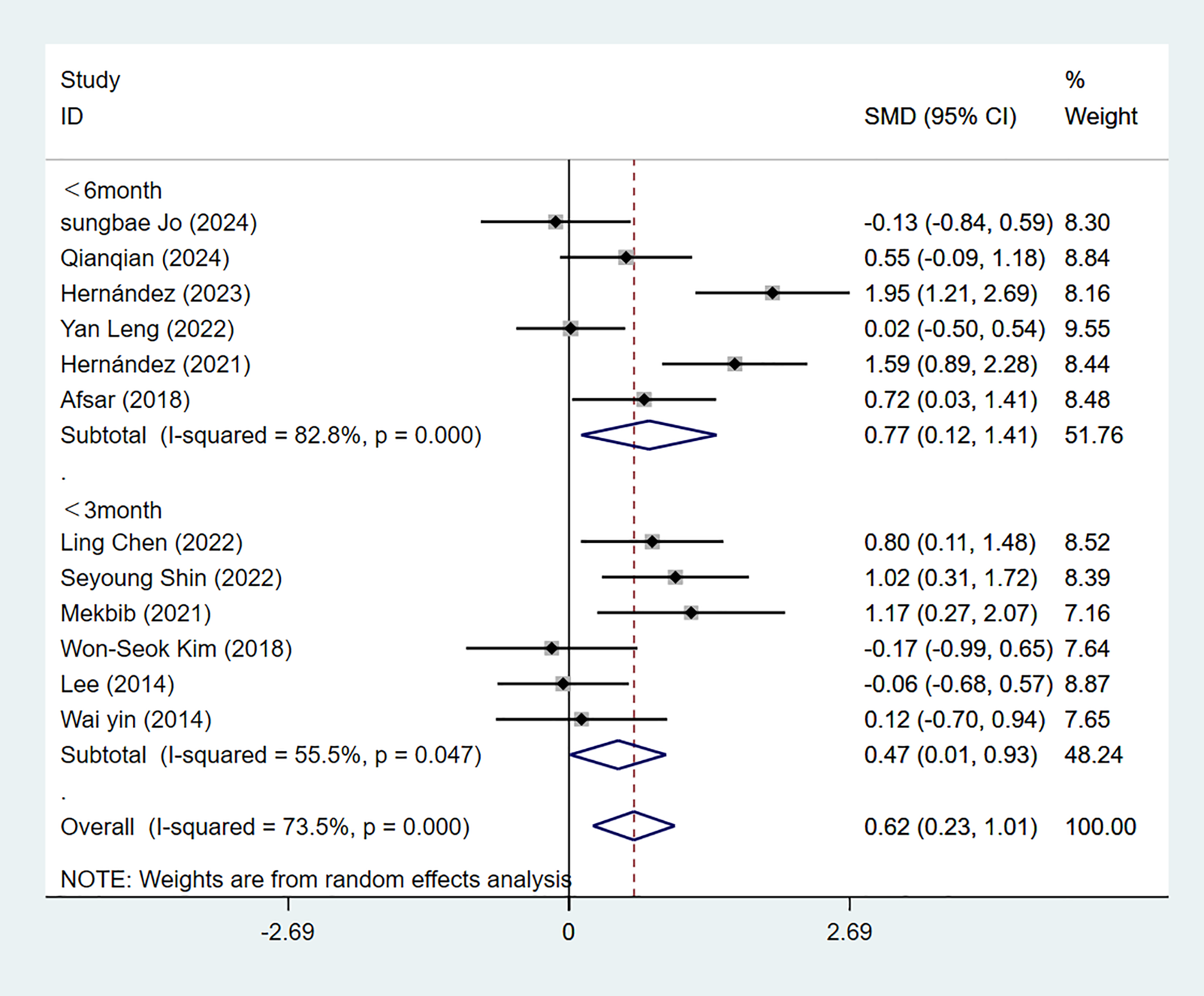

Supplement: Supplemental Information 11 [file peerj-14-21073-s011.png]

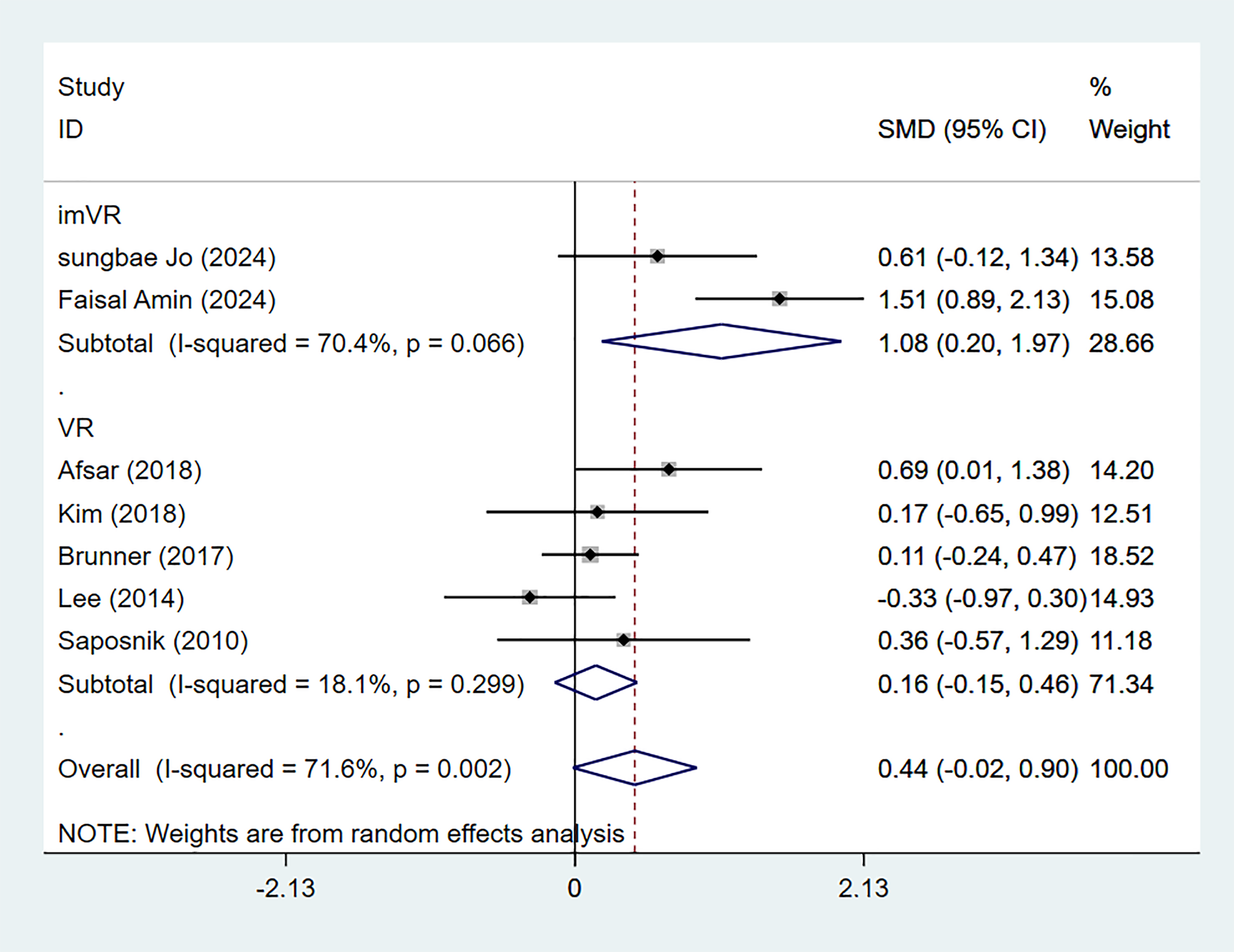

Supplement: Supplemental Information 12 [file peerj-14-21073-s012.png]

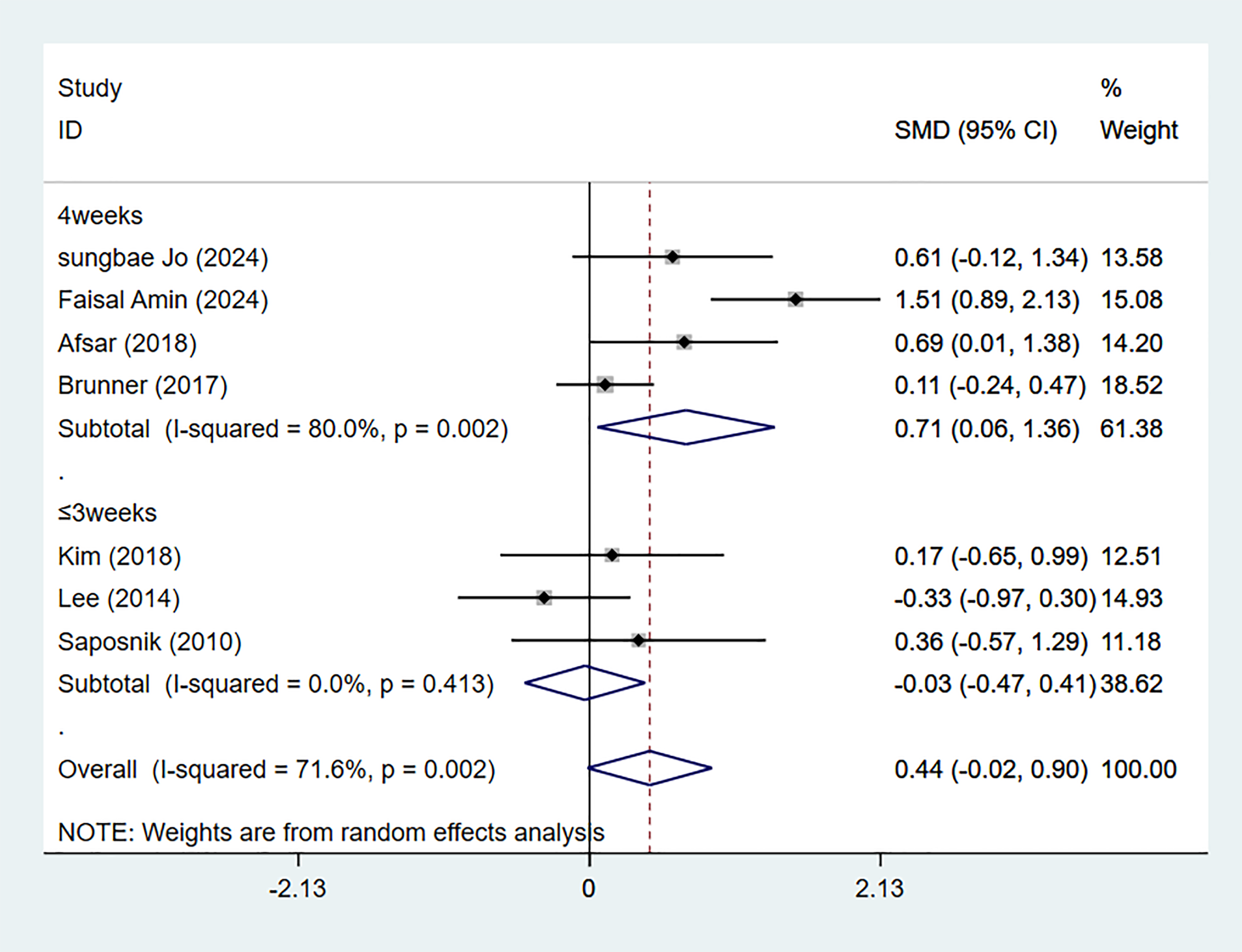

Supplement: Supplemental Information 13 [file peerj-14-21073-s013.png]

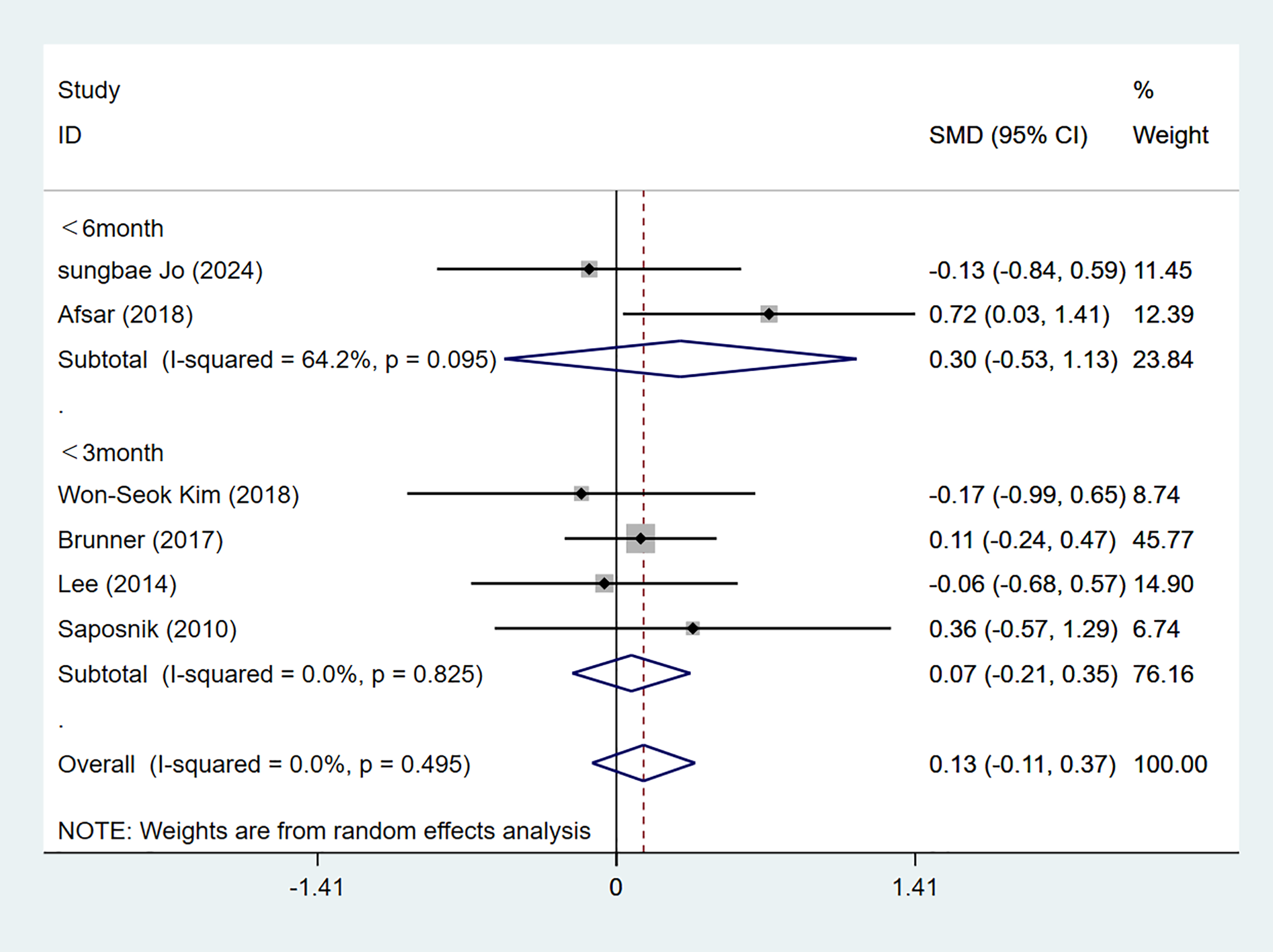

Supplement: Supplemental Information 14 [file peerj-14-21073-s014.png]

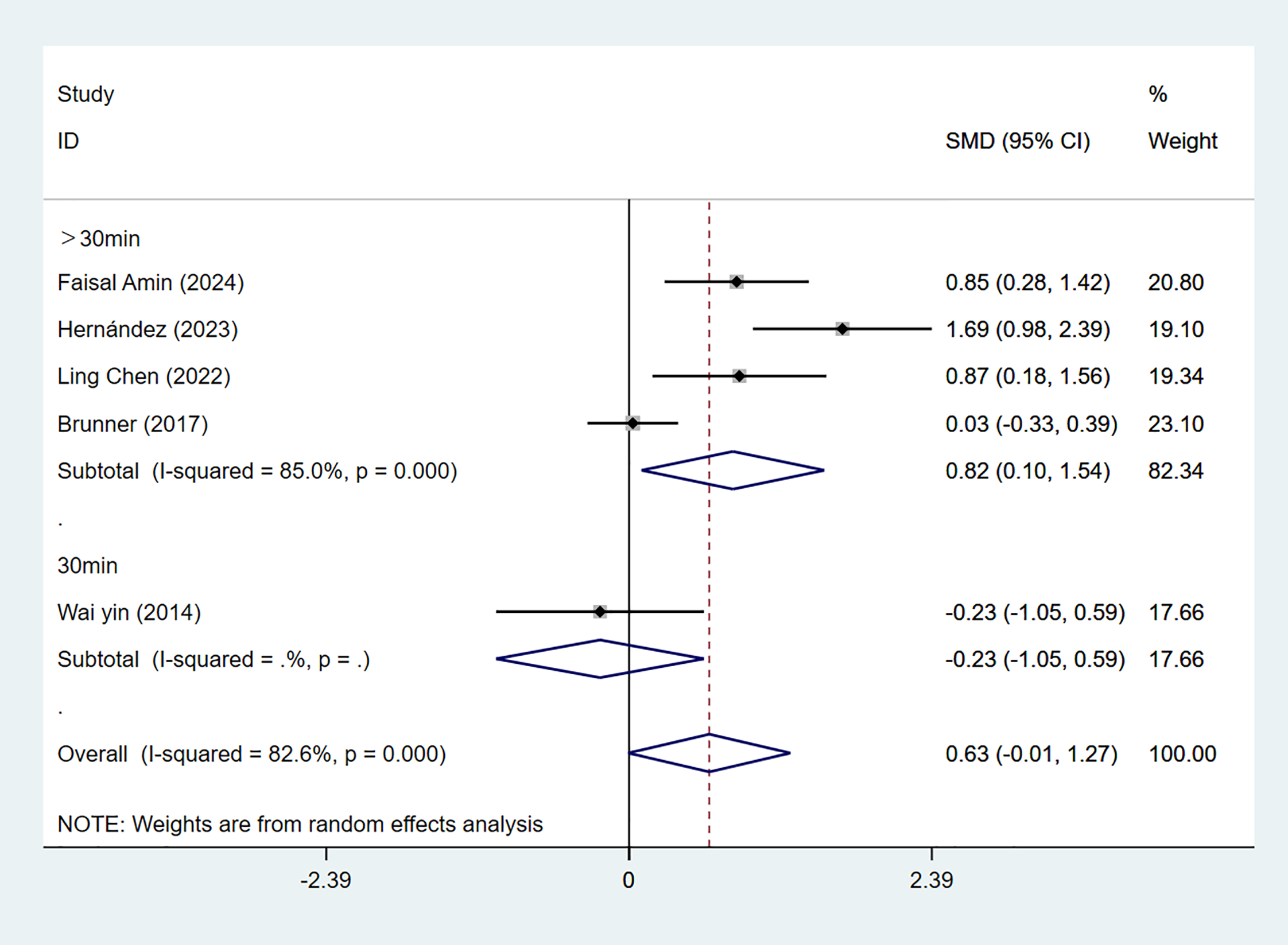

Supplement: Supplemental Information 15 [file peerj-14-21073-s015.png]

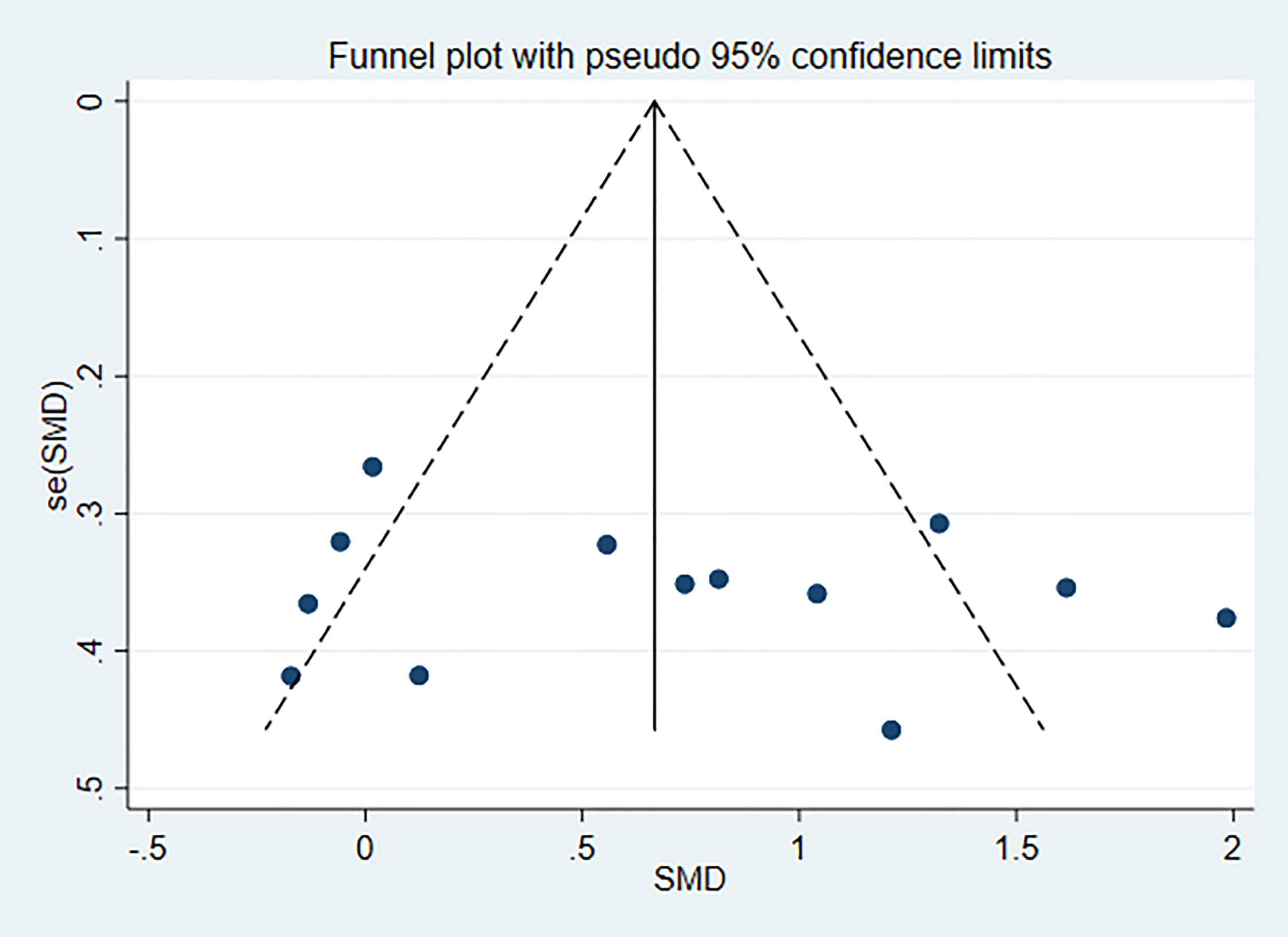

Supplement: Supplemental Information 16 [file peerj-14-21073-s016.png]

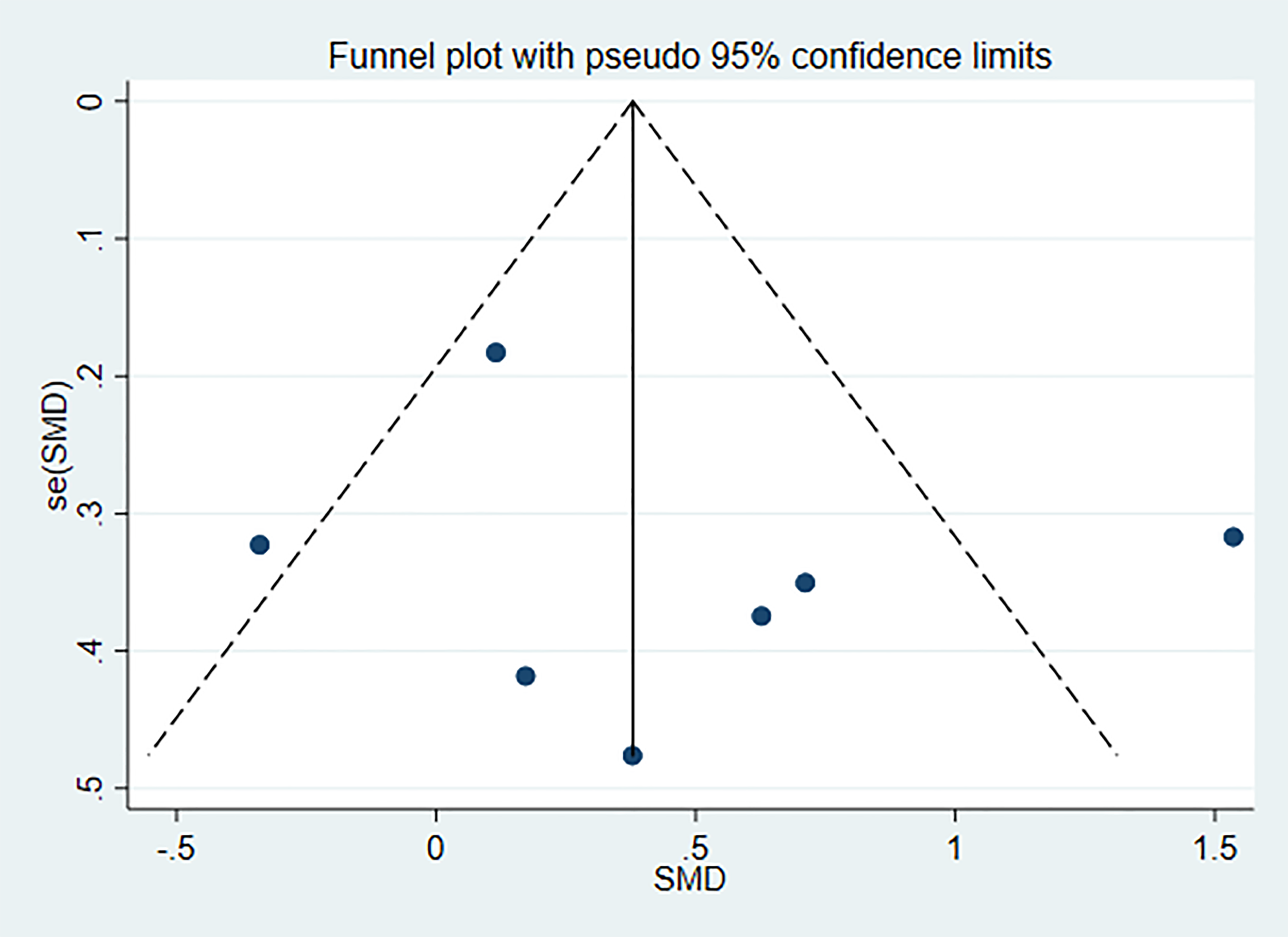

Supplement: Supplemental Information 17 [file peerj-14-21073-s017.png]

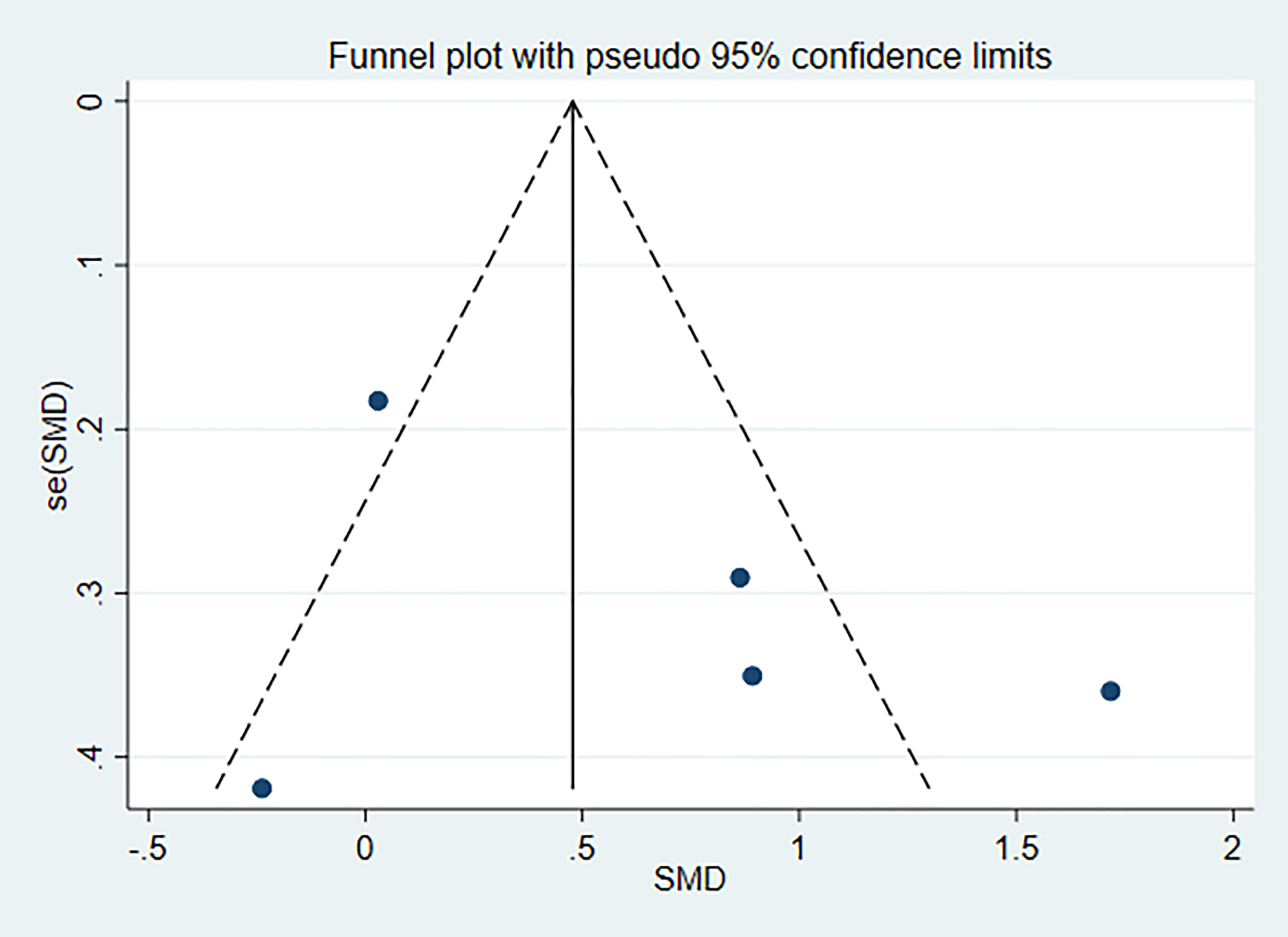

Supplement: Supplemental Information 18 [file peerj-14-21073-s018.png]
